# SNAIL Promotes Metastatic Behavior of Rhabdomyosarcoma by Increasing EZRIN and AKT Expression and Regulating MicroRNA Networks

**DOI:** 10.3390/cancers12071870

**Published:** 2020-07-11

**Authors:** Klaudia Skrzypek, Marta Kot, Paweł Konieczny, Artur Nieszporek, Anna Kusienicka, Małgorzata Lasota, Wojciech Bobela, Urszula Jankowska, Sylwia Kędracka-Krok, Marcin Majka

**Affiliations:** 1 Jagiellonian University Medical College, 31-008 Krakow, Poland; marta.kot@uj.edu.pl (M.K.); pawel.konieczny@outlook.com (P.K.); artur.nieszporek@gmail.com (A.N.); anna.kusienicka@gmail.com (A.K.); malgorzata.lasota@uj.edu.pl (M.L.); wojciech.bobela@gmail.com (W.B.); 2 Department of Transplantation, Institute of Pediatrics, Faculty of Medicine, Jagiellonian University Medical College, 30-663 Krakow, Poland; 3 Laboratory of Proteomics and Mass Spectrometry, Malopolska Centre of Biotechnology, Jagiellonian University, 30-387 Krakow, Poland; urszula.jankowska@uj.edu.pl; 4 Department of Physical Biochemistry, Faculty of Biochemistry, Biophysics and Biotechnology, Jagiellonian University, 30-387 Krakow, Poland; sylwia.kedracka-krok@uj.edu.pl

**Keywords:** rhabdomyosarcoma (RMS), tumor metastasis, SNAIL transcription factor, microRNA (miRNA), EZRIN, AKT kinase

## Abstract

Rhabdomyosarcoma (RMS) is a predominant soft tissue tumor in children and adolescents. For high-grade RMS with metastatic involvement, the 3-year overall survival rate is only 25 to 30%. Thus, understanding the regulatory mechanisms involved in promoting the metastasis of RMS is important. Here, we demonstrate for the first time that the SNAIL transcription factor regulates the metastatic behavior of RMS both in vitro and in vivo. SNAIL upregulates the protein expression of EZRIN and AKT, known to promote metastatic behavior, by direct interaction with their promoters. Our data suggest that SNAIL promotes RMS cell motility, invasion and chemotaxis towards the prometastatic factors: HGF and SDF-1 by regulating RHO, AKT and GSK3β activity. In addition, miRNA transcriptome analysis revealed that SNAIL-miRNA axis regulates processes associated with actin cytoskeleton reorganization. Our data show a novel role of SNAIL in regulating RMS cell metastasis that may also be important in other mesenchymal tumor types and clearly suggests SNAIL as a promising new target for future RMS therapies.

## 1. Introduction

Rhabdomyosarcoma (RMS) is a mesenchymal soft tissue tumor that causes death and morbidity predominantly in children and adolescents. Despite general improvement in the 5-year overall survival of pediatric RMS patients, for high grade tumors with metastatic involvement, the overall survival rate at 3 years is only 25–30% [1]. 6% of all cases with RMS display bone marrow (BM) metastasis, what significantly diminishes survival of the patients and makes them more prone to disease relapse and progression [2]. Therefore, understanding of the metastasis mechanisms is highly necessary and may help to find new treatment strategies in future. Important factors regulating the metastatic behavior of different tumor types, including RMS, are hepatocyte growth factor (HGF) and stromal-derived factor-1 (SDF-1) [3,4,5].

Histological analysis of tumors distinguishes two main subtypes of RMS, embryonal (ERMS) and alveolar (ARMS), with ARMS generally having a significantly worse prognosis [6]. The presence of PAX3-FOXO1 and PAX7/FOXO1 fusion genes and increased levels of MET receptors may cause the increased aggressiveness of ARMS tumors [7]. Mutations affecting the RAS-MAPK and PI3K-AKT pathways also affect RMS development and progression [1]. Among the critical regulators of RMS metastasis is the EZRIN protein, which acts as the actin filament-plasma membrane linker [8]. Crosstalk between different signaling pathways may create an integrated signaling network supporting the metastatic behavior of RMS.

Our previous studies identified the SNAIL family zinc finger 1 (SNAIL or SNAI1) transcription factor as a novel key regulator of RMS growth and differentiation [9,10]. We discovered noncanonical action mechanisms of SNAIL in RMS [9]. Importantly, the SNAIL level has been shown to be elevated in the ARMS tumor subtype, which is usually associated with a worse prognosis [11]. Moreover, the SNAIL level was significantly increased in RMS samples from patients displaying stages 2, 3, and 4 of the disease compared to those from patients with stage 1, suggesting an important role of SNAIL in RMS progression [9]. Furthermore, another member of the SNAIL family of zinc finger transcription factors, SNAI2, was identified by an integrative computational pipeline analysis as potential crucial factor in RMS growth [12].

SNAIL is best known as a regulator of epithelial to mesenchymal transition (EMT) through canonical regulation of the E-cadherin (CDH1) level [13,14,15]. It belongs to the SNAIL family of zinc finger transcription factors, which consists of 3 members: SNAIL (SNAI1), SLUG (SNAI2) and SMUG (SNAI3). SNAIL may act as a transcriptional repressor or as a gene activator by binding to target E-box sequences (CANNTG) [16] and recruiting histone deacetylases (HDACs) [13]. In addition to regulating protein expression levels, SNAIL is also a crucial regulator of microRNAs expression [17]. SNAIL silencing has been described to effectively suppress the growth and invasiveness of different tumor types [18]. SNAIL degradation is prevented in cancer cells due to stabilization of its level by the USP27X deubiquitinase [19]. SNAIL can also modulate myogenic differentiation by binding to the MYF5 promoter [9].

RMS originates from impaired differentiation of myogenic progenitors or mesenchymal stem cells (MSCs) [6]. Interestingly, SNAIL-deficient MSCs prematurely differentiate into osteoblasts or adipocytes [20], whereas in MSCs with a constitutively activated MET signaling pathway, SNAIL seems to act as a mediator of myogenic differentiation [21]. Moreover, SNAIL expression was demonstrated to be required for sarcomagenesis, as SNAIL controls the tumorigenic potential of MSCs [22]. In ARMS cells, SNAIL silencing completely abolished the growth of human tumor xenotransplants by upregulating myogenic differentiation [9], whereas in ERMS cells, SNAIL was identified as a mediator of the NOTCH pathway [23]. Similarly to SNAIL, myogenic transcription factors that may be important in RMS differentiation, such as MYOD and MYOG, bind to E-box sequences [24]. Furthermore, SNAIL can displace MYOD from E-box sequences that are associated with genes expressed during differentiation and it that way it may regulate RMS growth [9].

The metastatic process is very important for the dissemination of RMS cells and, consequently, for patients’ long-term prognoses. In epithelial tumors, SNAIL affects metastasis by regulating EMT [14]. The canonical action mechanism of SNAIL that promotes the metastatic process is the repression of E-cadherin expression by SNAIL binding to the E-cadherin promoter [13]. In addition, during the EMT process, cells acquire migratory properties by reorganization of the actin cytoskeleton and activation of the RhoA GTPase [25]. However, in mesenchymal tumors, different mechanisms might operate, and the mechanistic role of SNAIL in the metastasis of mesenchymal tumors is poorly understood. However, as in epithelial tumors, regulation of the actin cytoskeleton may also be a key process in the metastasis of mesenchymal tumor types.

## 2. Results

### 2.1. SNAIL Regulates the Metastatic Behavior of RMS Cells In Vivo and In Vitro

**Figure 1:**
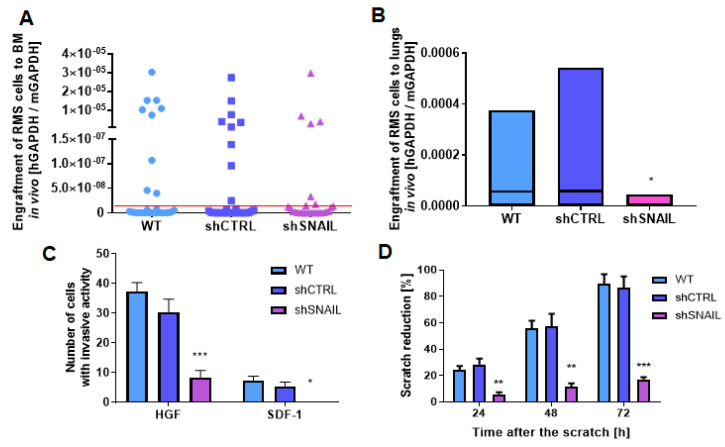

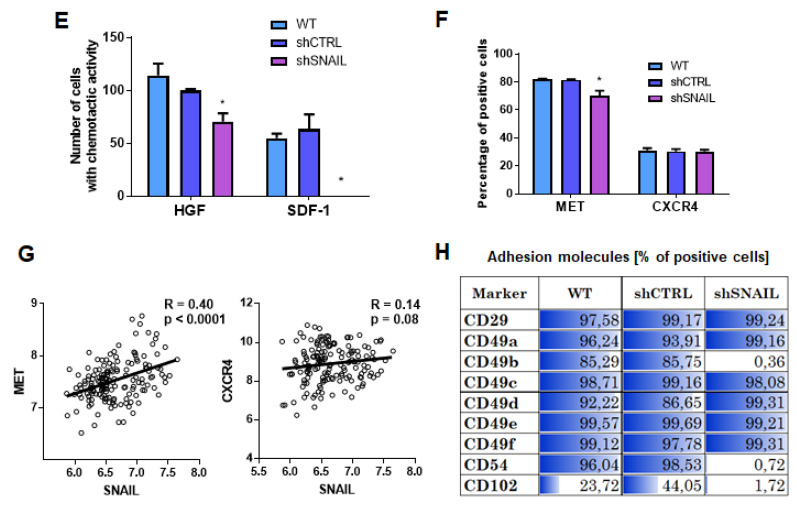
SNAIL regulates the motility of RMS cells in vitro and in vivo. (**A**) Stable SNAIL silencing by transduction with shRNA lentiviral vectors (shSNAIL) tended to inhibit the high level of engraftment of RH30 cells into murine bone marrow 7 days after intravenous implantation compared to that in WT cells and cells transduced with scrambled shRNA vectors (shCTRL). The presence of human cells was detected by qPCR and calculated as the ratio of the human to murine GAPDH levels with the ΔCt method. Data are presented as single points; *n* = 30 bone marrow samples (left and right) from 15 mice; red line discriminates bone marrow samples with high level of engraftment of human RMS cells. (**B**) Stable SNAIL silencing by transduction with shRNA lentiviral vectors (shSNAIL) inhibited the engraftment of RH30 cells into murine lungs 7 days after intravenous implantation compared to that in WT cells and cells transduced with scrambled shRNA vectors (shCTRL). The presence of human cells was detected by qPCR and calculated as the ratio of the human to murine GAPDH levels with the ΔCt method. Data are presented as a floating bar graph (min to max) with line at the mean; *n* = 14–15. (**C**) SNAIL-deficient cells displayed diminished invasion through Matrigel towards HGF (20 ng/mL) and SDF-1 (100 ng/mL), *n* = 3. (**D**) SNAIL deficient RH30 cells closed the gap in a scratch assay slower than control cells, *n* = 3. (**E**) In a chemotaxis assay SNAIL-deficient cells displayed diminished migration towards HGF (20 ng/mL) and SDF-1 (100 ng/mL), *n* = 3 (**F**) SNAIL silencing in RH30 cells diminished MET receptor levels but barely affected CXCR4 levels, as estimated by flow cytometry as the percentage of positive cells, *n* = 3. (**G**) SNAIL levels positively correlated with MET levels and slightly correlated with CXCR4 levels in 158 RMS samples from patients (Pearson correlation). Analysis was performed on data deposited in the GEO database with accession number: GSE92689. (**H**) SNAIL silencing in RH30 cells regulated the expression of adhesion molecules. The results are shown as the percentage of cells labeled with a Lyoplate Screening Panel (flow cytometry). * *p* < 0.05, ** *p* < 0.01, *** *p* < 0.001. Graphical data are presented as the means with SEMs.

In our previous studies, we stably silenced SNAIL with a mix of three different shRNA variants to study the effects of SNAIL on RMS cell growth and differentiation (Supplementary Figure S1) [9]. In the present study, we used that model to evaluate the effects of SNAIL on the metastatic behavior of RMS cells. RH30 cells were intravenously implanted into immunodeficient NOD-SCID mice. High level of engraftment of both RH30 WT and shCTRL (scrambled shRNA vector) cells was detected in nine out of 30 bone marrow samples per each group, whereas SNAIL silencing in RH30 cells reduced this number to five (Figure 1A). Furthermore, engraftment of SNAIL-deficient (shSNAIL) cells into the lungs in vivo was significantly impaired compared to that of both wild-type (WT) cells and cells modified with scrambled shRNA (shCTRL) (Figure 1B).

The observed impairment in the metastatic potential in vivo could be explained by the diminished invasion and motility of the cells. Indeed, in the invasion assay, SNAIL-deficient cells showed impaired migration through Matrigel (Figure 1C) after stimulation with SDF-1 and HGF, which promote RMS metastasis [4]. In the scratch assay, shSNAIL cells closed the gap in a monolayer slower than control cells (Figure 1D and Supplementary Figure S2). SNAIL silencing also resulted in diminished chemotaxis (Figure 1E) towards both SDF-1 and HGF. Interestingly, as suggested by the literature [26], SDF-1 is expressed in the murine lung (Supplementary Figure S3) and migration towards its gradient may be responsible for the tumor cells engraftment into the lungs in vivo. Expression of the HGF receptor MET was slightly diminished in SNAIL-deficient cells, whereas we did not observe any significant effect of SNAIL on the SDF-1 receptor CXCR4 (Figure 1F). Interestingly, bioinformatic analysis of microarray data deposited in the GEO database revealed a positive correlation between MET and SNAIL expression in 158 RMS tumor samples derived from patients but not between SNAIL and CXCR4 (Figure 1G). RH30 cells were also screened for expression of adhesion receptors that might be responsible for the cells’ engraftment in vivo: ICAMs and VLA integrins. VLA family members CD49a, b, c, d, e and f and CD29 were highly expressed in WT cells. Interestingly, SNAIL silencing completely diminished CD49b expression. Moreover, ICAM family receptors, i.e., CD54 (ICAM-1) and CD-102 (ICAM-2) were expressed in WT RH30 cells, whereas their expression in SNAIL-deficient cells was again diminished (Figure 1H). Thus, our data show an important role of SNAIL silencing in diminishing RMS cell metastatic properties by regulating their motility and adhesion properties.

### 2.2. EZRIN is a Crucial Mediator of the Metastatic Action of SNAIL in RMS Cells

**Figure 2:**
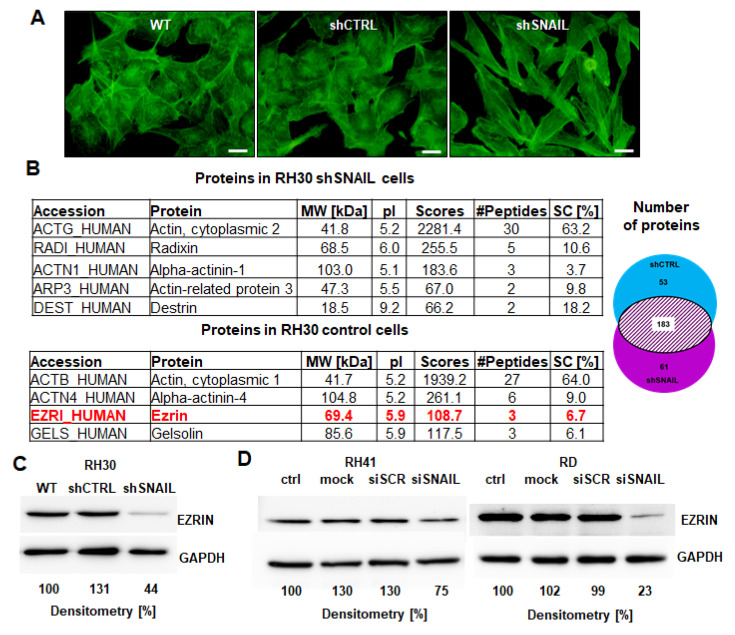

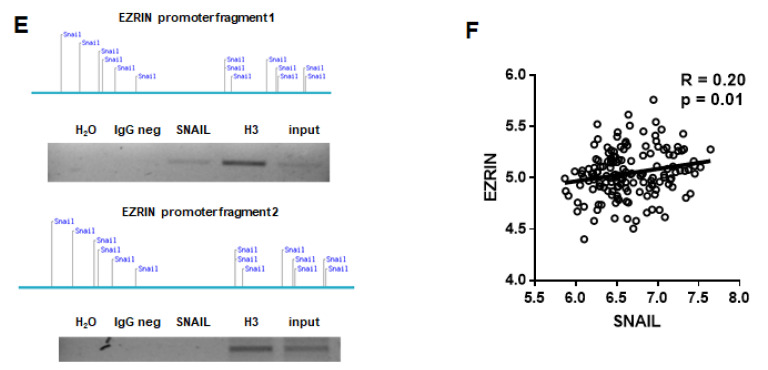
SNAIL regulates EZRIN expression in RMS cells. (**A**) SNAIL silencing in RH30 cells reorganized actin cytoskeleton (staining of RH30 cells with phalloidin conjugated with Alexa Fluor 488). White scale bar represents 50 μm. (**B**) SNAIL regulated proteins associated with the actin cytoskeleton in RH30 cells. Analysis of proteome by shotgun LC-MS/MS and MASCOT software revealed the expression of different proteins associated with the actin cytoskeleton in the cytoplasm of RH30 shSNAIL and shCTRL cells. Proteins for further consideration were identified based on at least 2 peptides. A total of 61 proteins were identified only in shSNAIL cells; 53, only in shCTRL cells; and 183, proteins in both cell types. Here only proteins related to actin cytoskeleton organization are shown. MW – molecular weight; pI –isoelectric point; scores – parameter describing the probability of the correct identification of protein; peptides – number of peptides in the analysis; SC [%]–obtained sequence coverage. (**C**) Stable SNAIL silencing in RH30 cells downregulated EZRIN expression at the protein level (Western blotting). The Western blot results show the representative image of three independent biological experiments. Densitometric analysis evaluated the ratio of EZRIN/GAPDH and was presented as percentage of control. (**D**) Temporal SNAIL silencing in RH41 and RD cells downregulated EZRIN expression at the protein level (Western blotting). The Western blot results show the representative image of three independent biological experiments. Densitometric analysis evaluated the ratio of EZRIN/GAPDH and was presented as percentage of control. (**E**) SNAIL bound to the *EZRIN* promoter in RH30 cells. Two fragments of *EZRIN* promoters (~1000 bp) were screened for putative SNAIL transcription factor binding sites and the results were validated by a ChIP assay. The images show one representative result of the ChIP assay. Proteins bound to DNA were immunoprecipitated with an anti-SNAIL antibody, negative IgG control, and positive histone H3 control. An input DNA served as control for analysis. Two fragments of the *EZRIN* promoter were amplified by PCR and visualized on agarose gels stained with ethidium bromide. (**F**) *SNAIL* levels were positively correlated with *EZRIN* levels in 158 RMS samples from patients (Pearson correlation). The analysis was done on data deposited in GEO database with accession number: GSE92689.

We evaluated the morphology of RMS cells and found strong reorganization of the actin cytoskeleton in shSNAIL RH30 cells (Figure 2A). Staining with phalloidin has not shown visible differences in fluorescence intensity between the examined lines. Significant differences were visible in the spatial organization of F-actin fibers, what can affect the cell adhesion and migration capacity [27]. In shSNAIL cells, the fibers are arranged parallel to each other, while in the control lines their arrangement is less ordered. Longitudinally oriented stress fibers in shSNAIL cells affect elongated morphology of these cells. Moreover, after SNAIL silencing the formation of membrane protrusions seems to be reduced. It has been proved that membrane protrusions (e.g. filopodia) are a key structure directly involved in cancer cell motility [28,29]. These findings can explain lower migration capacity of shSNAIL cells compared to control lines (WT and shCTRL).

Unbiased shotgun proteomic analysis of the cytoplasmic and nuclear fractions in RH30 cells revealed that SNAIL regulates the expression of several proteins associated with actin cytoskeleton organization including EZRIN (Figure 2B), which has been previously shown to be a key regulator of metastasis in RMS [8]. Diminished expression of EZRIN in shSNAIL RH30 cells was confirmed by Western blotting (Figure 2C). Moreover, when SNAIL was transiently silenced with siRNA in RH41 ARMS cells and RD ERMS cells, we observed downregulation of EZRIN expression (Figure 2D).

The eukaryotic promoter database describes two fragments of the *EZRIN* promoter: *EZR_1* chromosome [NC_000006.12]; strand [-]; position [158819364], and *EZR_2* chromosome [NC_000006.12]; strand [-]; position [158818235] [30]. Bioinformatic analysis demonstrated that the two fragments of the *EZRIN* promoter contain several putative SNAIL binding sites. The chromatin immunoprecipitation (ChIP) assay results revealed that SNAIL binds to one of the *EZRIN* promoter fragments (Figure 2E) and thus may directly regulate its expression. Interestingly, bioinformatic analysis of microarray data for 158 RMS patient samples deposited in the GEO database revealed a positive correlation between SNAIL and EZRIN expression levels (Figure 2F).

**Figure 3:**
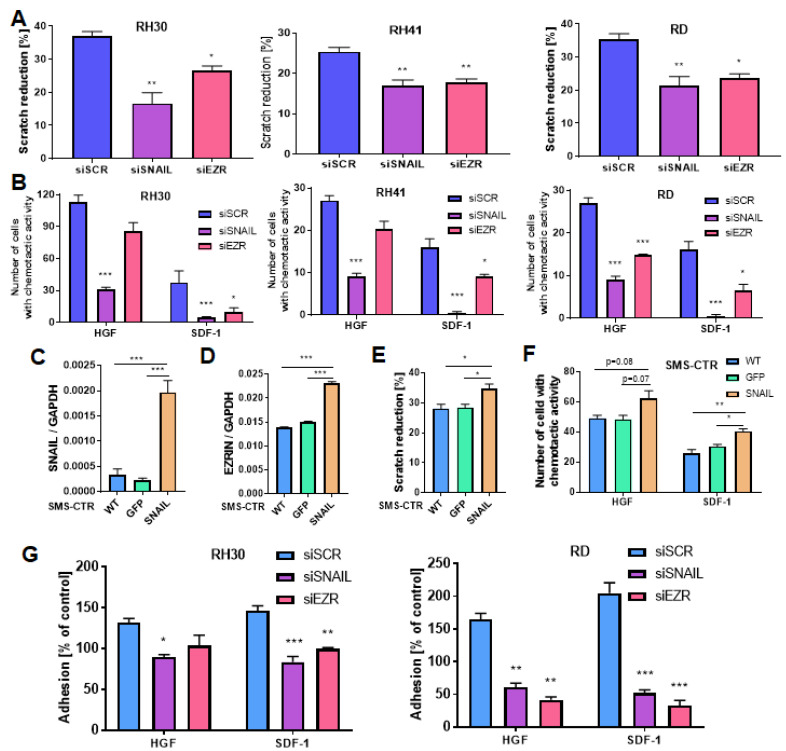

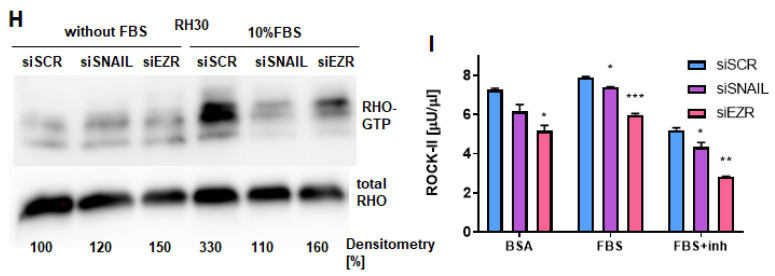
SNAIL and EZRIN are crucial regulators of migration, chemotaxis and adhesion of RMS cells to endothelial cells by affecting RHO activity. (**A**) SNAIL (siSNAIL) and EZRIN (siEZR) silencing with siRNA diminished the motility of RH30, RH41 and RD cells in a scratch assay. Cells were transfected three times every 3–4 days, and the assay was then performed; *n* = 3 (**B**) SNAIL silencing with siRNA inhibited chemotaxis of RH30, RH41 and RD cells towards HGF and SDF-1, whereas EZRIN silencing inhibited chemotaxis towards SDF-1 and HGF; *n* = 3. (**C**) SNAIL was overexpressed in SMS-CTR cells by transduction with lentiviral vectors, whereas control cells were modified with vectors encoding GFP; qPCR results; *n* = 2; WT- wild-type cells. (**D**) SNAIL overexpression in SMS-CTR cells upregulated EZRIN levels; qPCR results; *n* = 2. (**E**) SNAIL overexpression in SMS-CTR cells increased motility of the cells in a scratch assay; *n* = 3. (**F**) SNAIL overexpression in SMS-CTR cells increased chemotaxis towards SDF-1 and HGF; *n* = 3. (**G**) Both SNAIL and EZRIN silencing diminished the adhesion of RH30 and RD cells treated with HGF and SDF-1 to HUVECs pretreated with TNF-α. The graphs show representative results from three independent experiments, and the results were calculated as a percentage of the control. (**H**) Both SNAIL and EZRIN silencing by siRNA in RH30 cells inhibited RHO activation. RHO-GTP was detected by Western blotting. Representative Western blot images from two independent experiments after using the RHO Activation Assay Biochem Kit are shown. Densitometric analysis evaluated the ratio of RHO-GTP/total RHO and was presented as percentage of control. (**I**) SNAIL and EZRIN silencing with siRNA slightly diminished ROCK-II enzyme activity, as evaluated in protein lysates with a ROCK Activity Assay. Two independent experiments were performed with duplicate samples. * *p* < 0.05, ** *p* < 0.01, *** *p* < 0.001. Graphical data are presented as the means ±SEMs.

**Figure 4:**
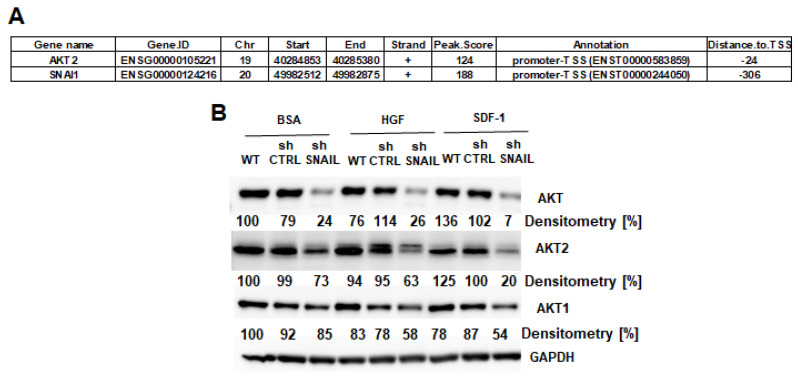

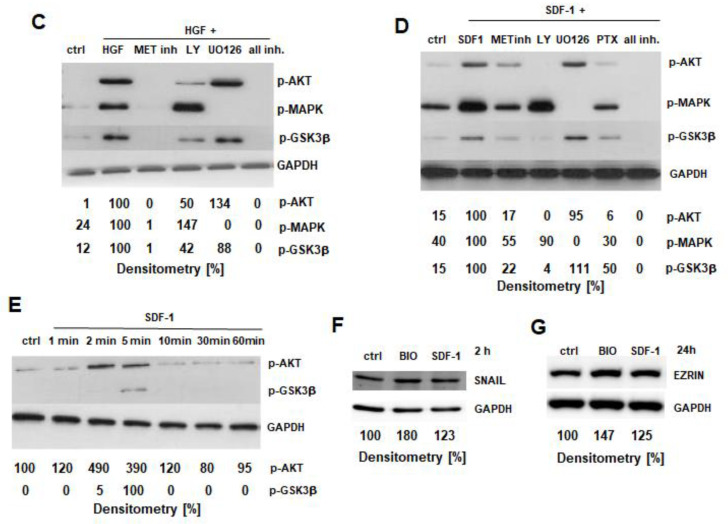
Mutual regulation of SNAIL and AKT expression in RMS cells. (**A**) SNAIL bound to the *AKT2* promoter and its own promoter, as shown by ChIP-seq analysis results in RH30 cells. (**B**) Stable SNAIL silencing in RH30 cells diminished total AKT and AKT2 and slightly decreased AKT1 levels under different treatment conditions (0.5% BSA, 20 ng/mL HGF, 100 ng/mL SDF1 and 10% FBS). Typical Western blot images of two independent biological experiments are shown. Densitometric analysis evaluated the ratio of protein of interest to GAPDH, presented as percentage of control. (**C**) HGF induced GSK3β phosphorylation via AKT kinase signaling pathway and not the MAPK kinase signaling pathway. RH30 cells were treated (20 ng/mL HGF, 5 min) together with the following inhibitors: 5 μM MET inhibitor, 30 μM LY294002 (PI3K-AKT inhibitor) or 30 μM UO126 (MAPK inhibitor). Negative control was starvation medium with 0.5% BSA without stimulants. Phosphorylation of AKT, MAPK and GSK3β was evaluated with GAPDH as loading control. Representative Western blot images of two independent biological experiments are shown. Densitometric analysis evaluated the ratio of protein of interest to GAPDH, presented as percentage of HGF-treated cells. (**D**) SDF-1 induced GSK3β phosphorylation via the AKT kinase signaling pathway and not the MAPK kinase signaling pathway. RH30 cells were treated (100 ng/mL SDF-1, 5 min) together with the following inhibitors: 5 μM MET inhibitor, 30 μM LY294002 (PI3K-AKT inhibitor), 30 μM UO126 (MAPK inhibitor) or 20 μg/mL PTX (signaling inhibitor via the Gα subunit). Negative control was starvation medium with 0.5% BSA and no stimulants. Phosphorylation of AKT, MAPK and GSK3β was evaluated with GAPDH as the loading control. Representative Western blot images of two independent biological experiments are shown. Densitometric analysis evaluated the ratio of protein of interest to GAPDH and was presented as percentage of SDF-1-treated cells. (**E**) SDF-1 induces phosphorylation of AKT and GSK3β in a sequential manner in RH30 cells. RH30 cells were treated with 100 ng/mL SDF-1. Negative control was starvation medium containing 0.5% BSA and no stimulants. Phosphorylation of AKT and GSK3β was evaluated with GAPDH as loading control. Western blot images of two independent biological experiments are shown. Densitometric analysis evaluated the ratio of protein of interest to GAPDH and is presented as % control (p-AKT) or % SDF-1-treated cells (p-GSK3β). (**F**) SDF-1 and BIO (inhibitor of GSK3β) induced expression of SNAIL protein 2 h after treatment. RH30 cells were treated with 100 ng/mL SDF-1 +1 μM BIO for two hours. SNAIL levels were evaluated with GAPDH as loading control. Western blot images of two independent biological experiments are shown. Densitometric analysis evaluated the ratio of SNAIL/GAPDH and was presented as percentage of control. (**G**) SDF-1 and BIO (inhibitor of GSK3β) induced EZRIN protein expression 24 h after treatment. RH30 cells were treated with 100 ng/mL SDF-1 and 1 μM BIO for 24 h. The EZRIN level was evaluated with GAPDH as a loading control. Western blot images of two independent biological experiments are shown. Densitometric analysis evaluated the ratio of genes of interests to GAPDH and was presented as percentage of control.

Subsequently, EZRIN and SNAIL were transiently silenced in three different RMS cell lines (ARMS: RH30 and RH41; EMRS: RD) (Supplementary Figure S4A,B) to verify their effects on the motility of these cells. Silencing of either SNAIL or EZRIN diminished the motility of RH30, RH41 and RD cells in a scratch assay (Figure 3A). SNAIL silencing also inhibited chemotaxis towards both HGF and SDF-1, whereas EZRIN silencing inhibited chemotaxis towards HGF in one cell line (RD) and chemotaxis towards SDF-1 in all cell lines (RH30, RH41, RD). However, the effect was less potent than in SNAIL-deficient cells (Figure 3B). Furthermore, to investigate not only effects of SNAIL silencing, but also its overexpression, SMS-CTR ERMS cells, which displayed low basal SNAIL level, were transduced with lentiviral vectors encoding SNAIL, whereas control cells were modified with vector encoding GFP (Figure 3C). SNAIL overexpression in SMS-CTR cell upregulated EZRIN levels (Figure 4B) and, accordingly, increased motility of the cells in a scratch assay (Figure 3E) and chemotaxis towards SDF-1 and HGF (Figure 3F).

### 2.3. Mutual Regulation of SNAIL and AKT Kinase Expression in RMS Cells

We performed ChIP-seq analysis of SNAIL binding sites in RMS cells, and the results revealed many significant SNAIL targets in RH30 cells (Supplementary Table S1). SNAIL transcription factor bound to the AKT2 kinase promoter and to its own promoter (Figure 4A and Supplementary Figure S5). These data suggested that SNAIL directly regulates the expression of AKT2 kinase in RMS cells. Additionally, Western blot analysis showed that SNAIL silencing diminished the expression of not only AKT2, but also AKT1 and total AKT (Figure 4B).

Subsequently, we evaluated the mechanism by which SNAIL can be regulated in RMS cells by HGF and SDF-1, the factors determining the metastatic capabilities of these cells. HGF and SDF-1 control GSK3β phosphorylation via the PI3K-AKT signaling pathway and not the MAPK pathway, as demonstrated by simultaneous treatment with HGF or SDF-1 and MET inhibitor, PI3K-AKT signaling inhibitor (LY294002), MAPK inhibitor (UO126) or pertussis toxin (PTX) (Figure 4C,D). LY294002 administration resulted in complete attenuation of AKT phosphorylation, accompanied by a decrease in GSK3β phosphorylation, whereas this effect was not observed after treatment with UO126 (Figure 4C,D). Moreover, PTX, an inhibitor of G-protein-coupled receptors (such as CXCR4) that impedes normal Gα subunit action, almost entirely abolished the stimulatory effect of SDF-1 (Figure 4D).

SDF-1 was demonstrated to induce the phosphorylation of AKT and GSK3β in sequential manner: first, AKT was phosphorylated, followed by GSK3β (Figure 4E). Interestingly, prolonged treatment with SDF-1 or the GSK3β inhibitor BIO resulted in slightly elevated SNAIL levels (Figure 4F) and, subsequently, slightly increased EZRIN level (Figure 4G). Our results demonstrate that SNAIL expression is regulated via the induction of GSK3β phosphorylation by the PI3K-AKT kinase signaling pathway and, moreover, identified SNAIL as an important regulator of AKT and EZRIN expression.

### 2.4. Inhibition of PI3K-AKT Signaling Diminishes Migration and Chemotaxis of RMS Cells

**Figure 5:**
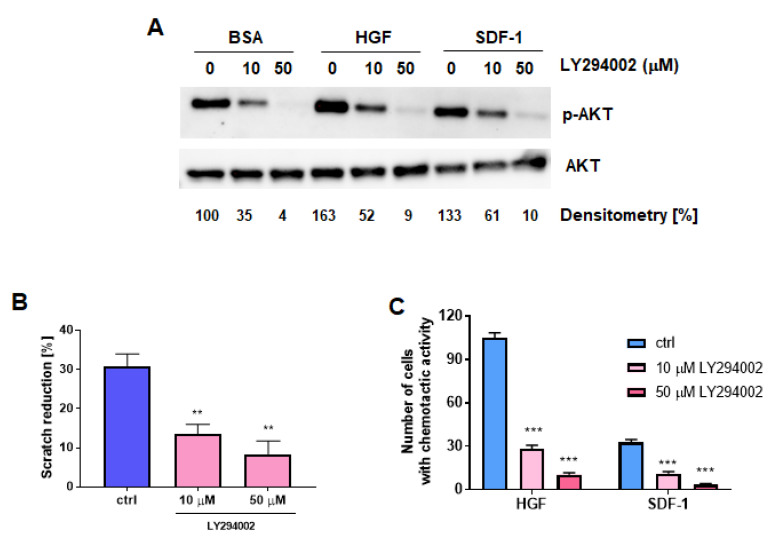
Inhibition of PI3K-AKT signaling diminishes migration and chemotaxis of RMS cells. (**A**) Treatment of RH30 cells with 10 and 50 μM LY294002 inhibited phosphorylation of AKT kinases under different treatments conditions (0.5% BSA, 20 ng/mL HGF, 100 ng/mL SDF-1). Cells were treated with the inhibitor 1 h before further experiments. Representative image of Western blots of two independent biological experiments is shown. Densitometric analysis evaluated the ratio of p-AKT to AKT and was presented as percentage of control. (**B**) Treatment of RH30 cells with 10 and 50 μM LY294002 inhibited the migration of the cells in a scratch assay; *n* = 3. (**C**) Treatment of RH30 cells with 10 and 50 μM LY294002 inhibited chemotaxis of RH30 cells towards 20 ng/mL HGF and 100 ng/mL SDF-1; *n* = 3. * *p* < 0.05, ** *p* < 0.01, *** *p* < 0.001. Graphical data are presented as the means ± SEMs. Alternatively, representative Western blot images of two independent biological experiments are presented.

To indirectly verify whether AKT kinase may be an important mediator of SNAIL-mediated RMS cell motility, WT RH30 cells were treated with LY294002, PI3K-AKT signaling inhibitor. We observed that a 10 μM concentration of the inhibitor partially inhibited AKT kinase phosphorylation, whereas 50 μM almost completely blocked its activation (Figure 5A). Moreover, both 10 and 50 μM LY294002 inhibited migration in a scratch assay (Figure 5B) and chemotaxis towards HGF and SDF-1 (Figure 5C). We discovered that the impaired AKT signaling mimicked the effects of SNAIL downregulation, what indirectly suggests that AKT kinase might be an important mediator of SNAIL action.

### 2.5. The SNAIL-miRNA Axis Regulates the Motility of RMS Cells

**Figure 6:**
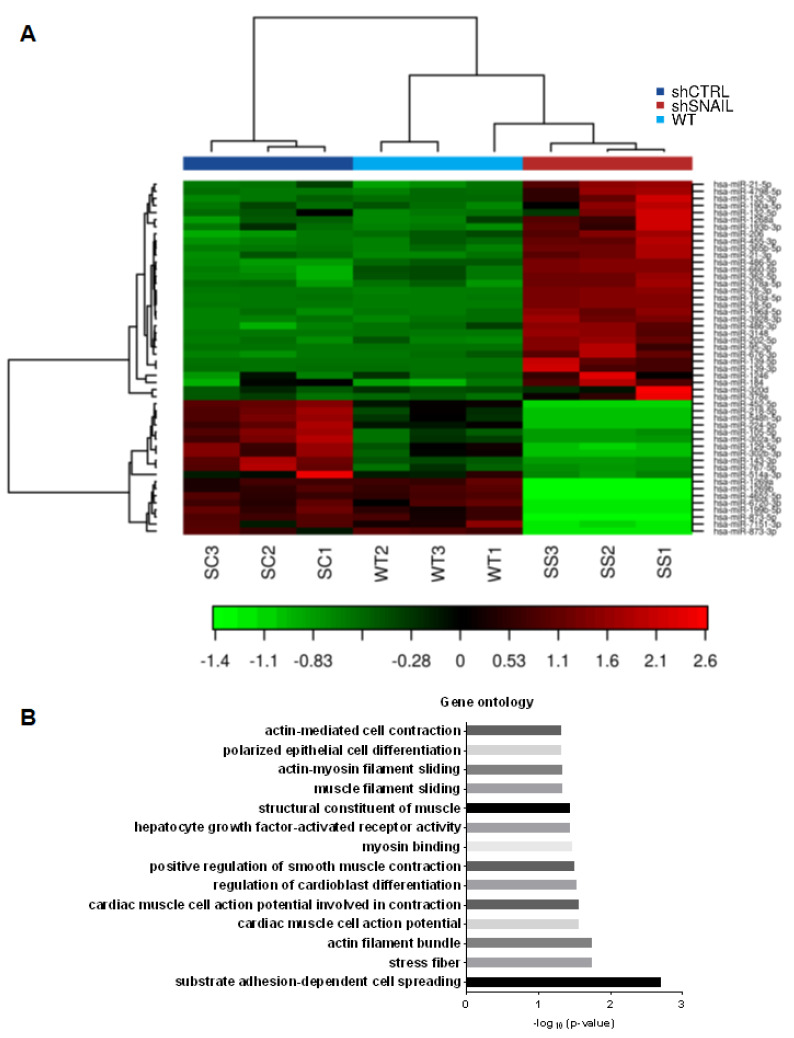
miRNAs are mediators of SNAIL action on cytoskeleton organization. (**A**) miRNA sequencing in RH30 WT, shCTRL (SC) and shSNAIL (SS) cells revealed that SNAIL silencing downregulates and upregulates many different miRNA sequences. These alterations were visualized as a heatmap via unsupervised hierarchical clustering by sample and miRNA. Clustering was performed on all samples, and the top 50 miRNAs with the highest coefficient of variation (CV) were identified based on the trimmed mean of M-values (TMM) normalized counts; *n* = 3. (**B**) GO enrichment analysis using Fisher’s test and the ‘Elim’ method revealed potentially significant biological processes and molecular functions associated with muscle and actin cytoskeleton structure in miRNA sequencing results; RH30 shSNAIL cells vs RH30 shCTRL cells, *p* < 0.05.

In addition to proteins, miRNAs may be the other important mediators of SNAIL action [17]. Therefore, the effect of SNAIL on the miRNA transcriptome was evaluated. Analysis of the miRNA transcriptome by next-generation sequencing showed that SNAIL silencing either upregulated or downregulated different miRNAs (Table 1 and Figure 6A and Supplementary Table S2). Gene ontology (GO) analysis of processes regulated by miRNAs revealed actin cytoskeleton reorganization and differentiation, as well as on HGF-activated receptor activity (Figure 6B), indicating that miRNAs are important mediators of SNAIL action. From the group of SNAIL-dependent miRNAs (Table 1 and Supplementary Table S2), four different candidate miRNAs were selected for further research. The two significantly upregulated and two downregulated miRNAs were selected for further analyses after verification of their levels using qPCR. The candidate miRNAs need to be expressed at a reasonable level, basing on TPM (tags per million) values (Supplementary Table S2).

**Figure 7:**
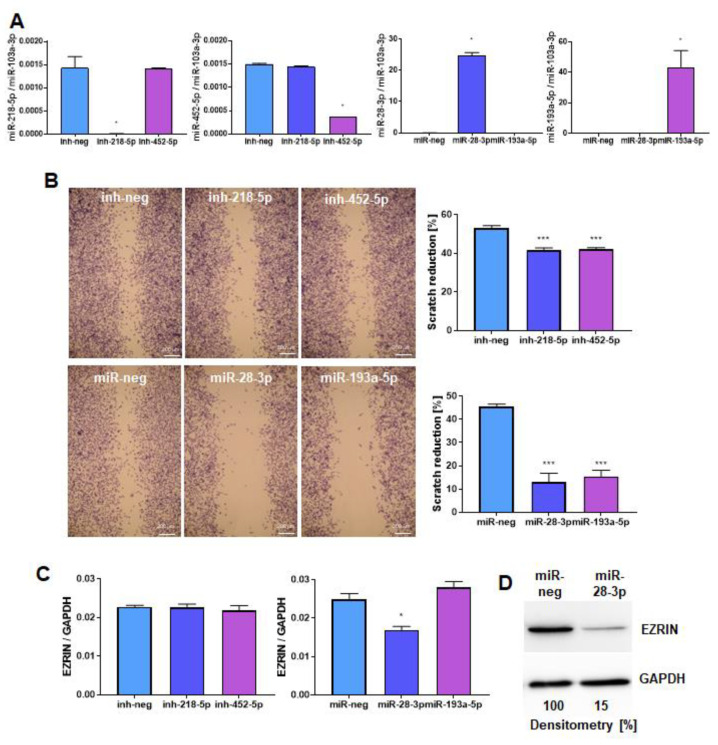

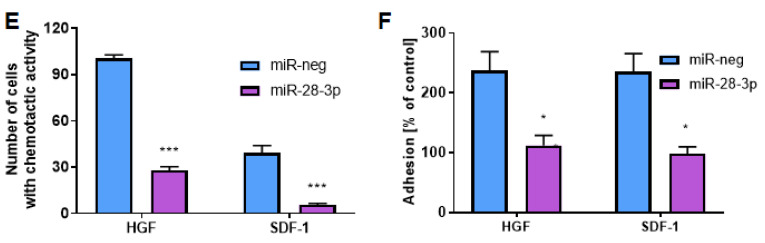
miR-28-3p is a mediator of SNAIL action on RMS cell motility and adhesion to endothelial cells. (**A**) SNAIL-dependent candidates miRNAs were either overexpressed by transfection with miRNA mimics (miR-28-3p and miR-193a-5p) or inhibited with miRNA inhibitors (miR-218-5p and miR-452-5p) in RH30 cells to evaluate whether the candidate miRNAs are important mediators of SNAIL action. The expression levels of miRNAs were evaluated three days after transfection by qPCR with the ΔCt method, and miR-103a-3p was used as a constitutively expressed miRNA control. The results are presented as the means of two independent experiments with duplicate samples. (**B**) Among the four miRNA candidates, miR-28-3p and miR-193a-5p regulated the most potently the motility of RH30 cells in a scratch assay. The results are presented as representative images of three independent experiments 24 h after scratching and graphs presenting the calculated percentage of scratch reduction; *n* = 3. White scale bar represents 200 μm. (**C**) Among the four miRNA candidates, only miR-28-3p was a regulator of *EZRIN* expression at the mRNA level in RH30 cells; *n* = 3. (**D**) miR-28-3p downregulated EZRIN at the protein level in RH30 cells (representative Western blot image of 2 independent biological experiments). (**E**) miR-28-3p overexpression diminished the chemotaxis of RH30 cells towards HGF and SDF-1; *n* = 3. (**F**) miR-28-3p overexpression in RH30 cells diminished the adhesion of RH30 cells treated with HGF and SDF-1 to HUVEC endothelial cells pretreated with TNF-α; *n* = 3. * *p* < 0.05, ** *p* < 0.01, *** *p* < 0.001. Graphical data are presented as the means ±SEMs. Alternatively, representative images are presented.

Among the selected candidates, miR-28-3p and miR-193a-5p were overexpressed in WT RH30 cells by transfection with miRNA mimics, as they were upregulated in SNAIL-deficient cells, whereas miR-218-5p and miR-452-5p were inhibited with miRNA inhibitors, as they were downregulated in SNAIL-deficient cells. The miRNAs expression levels after transfection were verified by qPCR (Figure 7A). The effect of those miRNAs on cell motility was screened by a scratch assay. miR-28-3p and miR-193a-5p inhibited the migration of RH30 cells the most potently (Figure 7B), indicating that they might be important mediators of SNAIL action.

Subsequently, we evaluated the effect of miRNA mimics and inhibitors on EZRIN levels. Only miR-28-3p downregulated EZRIN at the mRNA (Figure 7C) and at protein levels (Figure 7D). However, bioinformatic analysis did not show any binding sites for miR-28-3p in the *EZRIN* 3′-UTR, so we speculate that miR-28-3p regulates the EZRIN level indirectly. Overexpression of miR-28-3p inhibited chemotaxis towards HGF and SDF-1 (Figure 7E) and diminished the adhesion of RH30 cells to endothelial cells after treatment with HGF and SDF-1 (Figure 7F).

These results indicate that miR-28-3p may be an important mediator of the effects of SNAIL on migration, chemotaxis and adhesion through indirect modulation of EZRIN levels. The proposed mechanism of SNAIL regulation and its action in RMS is presented in graphical abstract.

## 3. Discussion

This study was undertaken to investigate the role of the SNAIL transcription factor in the regulation of RMS metastasis, since the action mechanism of SNAIL in mesenchymal tumors is not well understood. In our studies, we sought to identify different noncanonical action mechanisms of SNAIL in RMS metastasis that may also be important in other tumor types.

Previously, we showed that SNAIL is a key regulator of ARMS tumor growth and differentiation through functional repression of MYF5 and MYOD [9]. Here, we demonstrated that SNAIL affects RMS metastatic behavior by reorganizing the actin cytoskeleton and regulating intracellular pathways important for tumor cell metastasis. Actin cytoskeleton reorganization is also a key event in the acquisition of migratory properties by epithelial cancer cells undergoing EMT [25]. In addition, SNAIL was identified as a crucial factor involved in PI3K-AKT signaling and the EZRIN-RHO pathway that may be regulated by SDF-1 and HGF, factors that promote tumor cell metastasis.

An important pathway in RMS progression is the PI3K/AKT signaling pathway. Mutational activation of PI3K/AKT signaling has been previously associated with a clinically aggressive RMS subset [31]. We found that blocking this pathway in RMS cells results in decreased motility and chemotaxis towards HGF and SDF-1. Importantly, SNAIL was identified as a regulator of AKT expression by direct binding to the AKT2 promoter. Interestingly, SNAIL has been shown to enhance the binding of AKT2 to the E-cadherin (*CDH1*) promoter in epithelial cells and interference with AKT2 prevents SNAIL-mediated repression of the *CDH1* gene [32]. In addition, the literature suggests that AKT2 activates SNAIL and that a switch from the AKT1 to the AKT2 isoform is required for the induction of SNAIL expression [33]. Based on our results, we postulate the existence of a stimulatory loop between SNAIL and AKT that regulates RMS progression and metastasis. Our studies demonstrated that SDF-1 and HGF, which may stimulate tumor cells to form metastases, increase the SNAIL level by inducing GSK3β phosphorylation via the PI3K-AKT pathway. GSK3β regulation by AKT was previously demonstrated in different cell types [34]. The canonical pathway of AKT activation is initiated by G-protein-coupled receptors, such as CXCR4 and receptor tyrosine kinases, such as MET; subsequently, AKT mediates inhibitory phosphorylation of GSK3β S9 [35]. GSK3β regulates the SNAIL level by phosphorylation at two consensus motifs to affect the function of the protein either by ubiquitination or subcellular localization [36,37]. SNAIL can also bind to its own promoter, forming a feedback mechanism for its transcriptional regulation; this mechanism can be overridden in cells receiving potent stimulation of the PI3K pathway, which can activate SNAIL expression and subsequently induce EMT [38]. Our data confirm the existence of such feedback mechanisms in mesenchymal cells, since our ChIP-seq results showed the binding of SNAIL to its own promoter.

We have shown here for the first time that the EZRIN protein, which has been previously shown to be a key regulator of tumor metastasis [8,39], is a crucial mediator of SNAIL signaling pathways by acting as an actin filament-plasma membrane linker [8]. These results may be extrapolated to RMS patients because we found a positive correlation between *SNAIL* and *EZRIN* expression. EZRIN has been described as a metastatic determinant in many different tumor types [40], including mesenchymal tumors such as osteosarcoma [41].

The literature indicates a link between the EZRIN-dependent metastatic potential and the activity of the small GTPase RHO proteins [8]. Our studies demonstrated that both EZRIN and SNAIL silencing diminished the activity of RHO and its downstream mediator ROCK-II. RHO family GTPases and their downstream effector proteins – ROCK I and II – are often associated with enhanced invasive and metastatic phenotypes, as they are known regulators of the cytoskeleton and cell migration that are frequently overexpressed in different tumor types [42]. In addition, cell motility promoted by RHO signaling through ROCK was shown to require EZRIN localized in the direction of cell movement [43]. In oral cancer cells, SNAIL was previously associated with changes in RHO activity and phosphorylation of EZRIN-RADIXIN-MOESIN family, but no precise mechanism was described [44].

Our studies showed that SNAIL may regulate EZRIN expression by directly binding to its promoter. Via that mechanism SNAIL acts as an activator of EZRIN expression. SNAIL is usually considered as a transcriptional repressor, but it may also act as an activator [16], such as for MMP15 [45]. SNAIL can also potentiate enhancer activation by collaborating with different activators [46].

In this study we showed that in addition to directly regulating EZRIN, SNAIL can indirectly affect its level via miR-28-3p since bioinformatic analysis using TargetScanHuman 7.1 [47] and miRDB [48] did not reveal potential miR-28-3p binding sites in the 3′UTR region of *EZRIN* gene. miR-28-3p was identified as a SNAIL mediator in the motility, chemotaxis and adhesion of RMS cells. The important role of miR-28-3p in RMS was demonstrated for the first time. Previously, the role of miR-28 was suggested to be important in different tumor types, such as non-Hodgkin lymphoma [49] and colorectal cancer [50]. Our research also demonstrated that SNAIL silencing in RMS cells changes the miRNA transcriptome and that SNAIL-miRNA signaling regulates many different processes associated with actin cytoskeleton reorganization and cellular motility and differentiation. In addition to miR-28-3p, many different miRNAs are mediators of SNAIL action. Several studies in the literature describe SNAIL as a regulator of miRNA expression with implications for epithelial tumor progression [15,17], such as the interaction between miR-34 and SNAIL in the regulation of the EMT process [51].

Using an in vivo model, we showed diminished engraftment of SNAIL-deficient RMS cells in murine lungs after intravenous implantation. The metastatic capabilities of RMS cells may be regulated by the SDF-1 gradient. CXCR4–SDF-1 signaling regulates locomotion, chemotaxis, and adhesion [4]. The SDF-1 gradient was identified as an important factor in chemotaxis and adhesion of SNAIL-deficient RMS cells. Interestingly, our data suggest that SNAIL mediated motility of RMS cells is induced by the modulation of EZRIN levels rather than changes in CXCR4 expression. Moreover, SDF-1 may regulate SNAIL expression and thus may promote metastasis. HGF has also been shown to play an important role in RMS metastasis [7,21,52]. Our studies demonstrated that SNAIL regulates the level of the MET receptor, chemotaxis towards HGF and HGF-induced adhesion to endothelial cells. These results may also be extrapolated to patients suffering from RMS due to the correlation between SNAIL and MET receptor levels. Moreover, SDF-1 and HGF may also regulate the SNAIL level, as they induce rapid phosphorylation of AKT kinase and GSK3β [53].

The diminished engraftment of SNAIL-deficient RMS cells in murine lungs may be explained by the inhibited adhesion of RMS cells to activated endothelial cells. This process is affected by SNAIL through its regulation of EZRIN, miR-28-3p levels and adhesion molecules. In this study, we showed that SNAIL silencing completely blocked CD49b and ICAM-1 (CD54) expression and profoundly diminished ICAM-2 (CD102) levels. Integrin adhesion to the ECM provides the traction required for tumor cell invasion [54]. Importantly, CD49b (VLA-2 integrin) expression plays major roles in the post extravasation movement of RMS cells [55].

In conclusion, our data demonstrate a novel mechanism by which the SNAIL transcription factor regulates RMS metastasis. We showed that SNAIL forms an integrated signaling network supporting the progression of RMS and that its expression may be induced by factors attracting tumor cells to form metastases, such as SDF-1 and HGF. Regulatory mechanisms mediated by SNAIL crosstalk between AKT kinase, EZRIN, RHO-GTPase and miRNAs modulate tumor cell motility, adhesion and actin cytoskeleton organization, supporting the progression and metastasis of RMS (see the Graphical Abstract). We believe that these newly discovered mechanisms may also be important in other tumor types.

## 4. Materials and Methods

### 4.1. Cell Culture

RMS cell lines (RH30, RH41, RD, SMS-CTR) were kindly provided by Dr. PJ Houghton (Center for Childhood Cancer, Columbus, OH, USA). The cells were cultured in DMEM high-glucose medium (Lonza Group Ltd., Basel, Switzerland) supplemented with 10% fetal bovine serum (FBS, EURx, Gdansk, Poland) and 50 μg/mL gentamicin (Lonza) at 37 °C, 5% CO_2_ and 95% humidity.

Human umbilical vein endothelial cells (HUVEC) have been ordered from Becton Dickinson Biosciences. They were cultured in endothelial cell growth medium (PromoCell, Heidelberg, Germany), with endothelial cell growth supplement (PromoCell).

The cell lines were routinely tested for *Mycoplasma* spp. contamination using by MycoAlert™ Mycoplasma Detection Kit (Lonza). RMS cell line authentication was performed by STR profiling using AmpFlSTR SGM PLUS Kit (Applied Biosystems, Foster City, CA, USA) and sequencing apparatus ABI Prism 310 Genetic Analyser (Applied Biosystems) according to the manufacturer’s protocol.

### 4.2. Treatments of the Cells

For evaluation of expression and phosphorylation of kinases, RH30 cells were examined at 70% confluency. They were starved overnight in DMEM medium with 0.5% BSA. Subsequently, they were treated with 20 ng/mL HGF (Peprotech, London, UK) for 10 min, 100 ng/mL SDF-1 for 2–5 min in starving medium and 10% FBS for 30 min. Subsequently, protein was isolated with MPER buffer, as described below.

For experiments with LY294002 inhibitor (Merck, Darmstadt, Germany) cells were pretreated with 10 or 50 μM LY294002 one hour before further treatments with HGF, SDF-1 and FBS. In scratch assay and chemotaxis experiments the inhibitor was added one day before the experiment and it was present for the whole experiment.

The other inhibitors used in the experiments with treatment of the cells with SDF-1 or HGF were following: pertussis toxin (PTX) 20 μg/mL (Sigma-Aldrich, Darmstadt, Germany) was added 1.5 h before stimulation, UO126 30 μM (Merck) was added 1 h before stimulation, MET inhibitor 5 mM (Merck) was added 16 h prior to stimulation, BIO 1 μM was added for different time periods between 2 and 24 h.

### 4.3. Production of Viral Vectors and Transduction of Cells

RH30 cells were transduced with shRNA Lentiviral Particles targeting SNAIL and control lentiviral particles (Santa Cruz Biotechnology, Santa Cruz, CA, USA), as described previously [9]. shRNA Lentiviral Particles consisted of three different shRNA sequences targeting SNAIL.

Lentiviral particles encoding GFP-P2A-SNAIL (GFP-P2A-SNAIL @pLenti6/UbC) and GFP (GFP@pLenti6/UbC) were produced using the Vira Power Lentiviral Expression System (Invitrogen, Carlsbad, CA, USA), as previously described [9,21,56]. SMS-CTR cells were transduced with lentiviral vectors (at MOI = 10) in the presence of 6 μg/mL polybrene (Sigma-Aldrich). After 72 h the cells were subject to selection with 2.5 μg/mL blasticidin (InvivoGen, Toulouse, France) for 2 weeks.

### 4.4. Transfection with siRNA

RH30, RH41 and RD cells were transfected with 20 nM siRNA against SNAIL (combination of two Silencer Select siRNA ID variants: s13185 and s13187, Ambion Inc., Austin, TX, USA) or against EZRIN (combination of two Silencer Select siRNA ID variants: s14795 and s14797) or scrambled control siRNA (Silencer Select Negative Control #1 siRNA, cat. 4390844, Ambion) using Lipofectamine RNAiMAX (Invitrogen) transfection reagent according to vendor’s protocol. Protein level was verified three days after transfection. Three subsequent transfections were performed every 3–4 days and after that time cells were seeded for the experiments involving scratch assay and chemotaxis.

### 4.5. Transfection of Cells with miRNA Precursors and Inhibitors

RH30 cells were transfected with 30 nM mirVana™ miRNA Mimic: hsa-miR-28-3p (ID: MC12933), hsa-miR-193a-5p (ID: MC1178), Negative Control 1 or alternatively with mirVana™ miRNA Inhibitor: hsa-miR-218-5p (MH10328), hsa-miR-452-5p (ID: MH12509), Negative Control 1 using using Lipofectamine RNAiMAX (Invitrogen) transfection reagent according to vendor’s protocol. RNA was isolated 48–72 h after transfection. The cells were seeded for further experiments 48 h after transfection.

### 4.6. DNA and RNA Isolation and Reverse Transcription

Total RNA was extracted using the GeneMATRIX Universal RNA/miRNA Purification Kit (EURx) or mirVana miRNA Isolation Kit (Ambion), according to the manufacturer’s protocol. Reverse transcription of mRNA was performed using MMLV reverse transcriptase (Promega, Madison, WI, USA) according to the vendor’s protocol. Reverse transcription of miRNA was performed using the Universal cDNA Synthesis Kit (Exiqon, Vedbaek, Denmark) or miRCURY LNA RT Kit (Qiagen, Hilden, Germany), according to the manufacturer’s protocol.

### 4.7. Quantitative Real-Time PCR

Gene expression was determined by qRT-PCR analysis using QuantStudio™ 7 Flex Real-Time PCR System (Applied Biosystems), Blank qPCR Master Mix (EURx) and the indicated Taq-Man probes (Applied Biosystems): human: *GAPDH* (Hs99999905_m1), *SNAI1* (Hs00195591_m1) and *EZRIN* (Hs00931653_m1). The mRNA expression level for all of the samples was normalized to the housekeeping gene *GAPDH*, using the 2^−ΔCt^ method.

For the evaluation of miRNA expression by quantitative real-time PCR, SYBR Green qPCR Master Mix (EURx) with LNA™ PCR primer set (Exiqon) or miRCURY LNA miRNA PCR Assay (Qiagen) for human miR-28-3p, miR-193a-5p, miR-218-5p, miR452-5p and miR-103a-3p were used. The miRNAs expression levels were quantified using the 2^−ΔCt^ method, using miR-103a-3p as a relative control.

### 4.8. MicroRNA Sequencing

Library preparation, next generation sequencing and analysis of data were performed at Exiqon Services in Denmark. A total of 1 μg of total RNA was converted into microRNA NGS libraries using NEBNEXT library generation kit (New England Biolabs Inc., Ipswich, MA, USA) according to the manufacturer’s instructions. Each individual RNA sample had adaptors ligated to its 3′ and 5′ ends and converted into cDNA. Then the cDNA was pre-amplified with specific primers containing sample specific indexes. After 15 cycle pre-PCR the libraries were purified on QiaQuick columns and the insert efficiency evaluated by Bioanalyzer 2100 instrument on high sensitivity DNA chip (Agilent Inc., Santa Clara, CA, USA) The microRNA cDNA libraries were size fractionated on a LabChip XT (Caliper Inc., Waltham, Massachusetts, USA) and a band representing adaptors and 15–40 bp insert excised using manufacturer’s instructions. Samples were then quantified using qPCR and concentration standards. Based on quality of the inserts and the concentration measurements the libraries were pooled in equimolar concentrations (all concentrations of libraries to be pooled are of the same concentration). The library pools were finally quantified again with qPCR and optimal concentration of the library pool used to generate the clusters on the surface of a flow cell before sequencing using v2 sequencing methodology according to the manufacturer instructions (Illumina Inc., San Diego, CA, USA).

Samples were sequenced on the Illumina NextSeq 500 system. The system uses quality score binning enabling a more compact storage of raw sequences. Using only eight levels (Levels: No call, 6, 15, 22, 27, 33, 37, 50) of quality method has been tested and found to virtually loss-less. Average number of reads per sample was 7.5mio. Number of sequencing cycles (read length) was 50 nt, single-end read. The differential expression analysis was done using TMM (the trimmed mean of M-values normalization method) in the EdgeR statistical software package (Bioconductor, http://www.bioconductor.org/). Exiqon Services also performed TPM (tags per million) normalization. MicroRNA NGS data were deposited in Gene Expression Omnibus (GEO) under accession number GSE100114.

### 4.9. Western Blotting

Total extracts of protein were isolated with M-PER lysing buffer (Pierce, Rockford, IL, USA) as described previously [21], whereas nuclear and cytoplasmic fractions of protein, were isolated using the Nuclear Extract Kit (Active Motif, La Hulpe Belgium) according to the manufacturer’s protocol. The protein concentration was measured using the Bio-Rad Protein Assay (Bio-Rad, Hercules, CA, USA) according to the vendor’s protocol. Western blotting was performed using the anti-GAPDH rabbit mAb (14C10; #2118; Cell Signaling Technology, Leiden, The Netherlands), the anti-histone H3 (ab1791; Abcam, Cambridge UK), the anti-SNAIL mouse mAb (L70G2; #3895; Cell Signaling), anti-EZRIN (3C12, Santa Cruz Biotechnology), anti-phospho-AKT (Ser473) rabbit mAb (#9272, Cell Signaling, Danvers, MA, USA), anti-AKT rabbit pAb (#9272, Cell Signaling), anti-AKT2 mouse mAb (#5239, Cell Signaling), anti-AKT1 rabbit mAb (#2938T, Cell Signaling), anti-phospho-p44/42 MAPK (9106S, Cell Signaling), anti-phospho-GSK-3β (Ser9) rabbit mAb (#9336, Cell Signaling), anti-GSK-3β rabbit mAb (#9315, Cell Signaling), and secondary anti-rabbit and anti-mouse antibodies conjugated with horseradish peroxidase (HRP, Santa Cruz Biotechnology). Proteins were separated by electrophoresis in a 12% resolving sodium dodecyl sulfate–PAGE gel, and the fractionated proteins were transferred into a PVDF membrane (BioRad). Chemiluminescent signals were developed using (SuperSignal™ West Pico PLUS Chemiluminescent Substrate (Thermo Scientific) and ChemiDoc MP Imaging System (Bio-Rad) or using developing films. Western blot results are presented as representative images of two or three independent biological experiments. Densitometric analysis of each Western blot image was performed using ImageLab software (BioRad). The ratio of the adjusted volume band of the gene of interest to the constitutive gene was evaluated, and subsequently results were presented as percentage of control.

### 4.10. RHO and ROCK-II Enzymes Activity

RHO protein activity was evaluated using Rho Activation Assay Biochem Kit (Cytoskeleton, Inc., Denver, CO, USA), according to vendor’s protocol. For the experiment, RH30 cells were transfected with siRNA for the three times, then starved in 0.5% FBS for 24 h, then 0% FBS for a further 24 h and then treated with 10% FBS 15 min before lysis according to vendor’s protocol. The results were analyzed by Western blot.

Rho-associated Kinase (ROCK) Activity Assay (Merck Millipore, Darmstadt, Germany) was used to evaluate ROCK-II enzyme activity in protein lysates, according to vendor’s protocol. Inhibitor from the kit was used to evaluate assay specificity.

### 4.11. Analysis of Subproteomes of Nuclear and Cytoplasmic Fractions

Cytoplasmic and nuclear protein fractions were isolated by the ProteoExtract Kit (Calbiochem, San Diego, CA, USA). Protein samples were diluted with 8 M urea in 50 mM ammonium bicarbonate, reduced with DTT, alkylated with iodoacetamide and digested with trypsin on 30 kDa cut-off filter (Vivacon 500, Sartorius Stedim, Goettingen, Germany) using filter-aided sample preparation (FASP) procedure described previously [57].

Peptides were further analyzed by use of shotgun LC-MS/MS technique using reversed-phase liquid chromatography (RP-LC) system (UltiMate 3000RS LCnanoSystem, Dionex, Sunnyvale, CA, USA) coupled with a quadrupole time-of-flight mass spectrometer (micrOTOF-Q II, Bruker Daltonics, Bremen, Germany).

The RP-LC system consisted of a desalting trap column (75 μm × 2 cm, C_18_ material, 3 μm, 100 Å) and an analytical column (75 μm × 50 cm, C_18_ material 2 μm, 100 Å) with a nanoflow solvent delivery. The LC-MS/MS data were acquired by online analysis of peptides eluted with a 90 min gradient ranging from 2% to 40% acetonitrile in 0.05% formic acid/water at a 300 nl/min flow rate. MS/MS data were obtained by targeting 5 precursor ions in the scan range of m/z 50 to 2500 Da. The raw data were processed by Data Analysis 4.1 (Bruker Daltonics) and searched against Swiss Prot_201407 database with taxonomy restriction to Homo sapiens (20,284 sequences) using MASCOT search engine (v.2.3.0) embedded into ProteinScape 3.0 (Bruker Daltonics). The following parameters were used for the search: trypsin with maximum one missed cleavage, precursor and product ions mass tolerance were respectively ±20 ppm and ±0.05 Da; fixed modification—carbamidomethylation (C); variable modifications—oxidation (M), deamidation (NQ). Proteins below the 1% false discovery rate were considered. The complete analysis was composed of two LC-MS/MS runs. On the basis of the first run, a scheduled precursor list (SPL) was generated and used as an exclusion list during second LC-MS/MS analysis. A compilation of obtained two search results was performed with the ProteinScape 3.0 platform.

The mass spectrometry proteomics data have been deposited to the ProteomeXchange Consortium via the PRIDE [58] partner repository with the dataset identifier PXD006711 and 10.6019/PXD006711. The data include Bruker mass spectrometer output files (.baf) and PRIDE XML files generated from Mascot DAT files with the use of PRIDE Converter 2 [59].

### 4.12. Bioinformatic Analysis

Ezrin promoter fragments were found in the Eukaryotic promoter database [30]. EPD describes two fragments of *EZRIN* promoter: *EZR_1* 9Chromosome [NC_000006.12]; Strand [-]; Position [158819364]) and *EZR_2* (Chromosome [NC_000006.12]; Strand [-]; Position [158818235]). Two fragments of the promoter of *EZRIN* (~1000 bp) was screened for putative SNAIL transcription factor binding sites using a TF prediction tool called ConSite (http://consite.genereg.net/). The search for putative TF binding sites was performed at 80% cutoff [60]. The results were then compared with other TF prediction tools.

### 4.13. Chromatin Immunoprecipitation (ChIP) Assay

ChIP was performed using SimpleChip Enzymatic Chromatin IP Kit (Cell Signaling Technology) according to the manufacturer’s protocol. For ChIP assays 10 µg of goat antibody against SNAIL (cat. AB-108-C; R&D Systems, Minneapolis, MN, USA), 10 µg of the positive control histone H3 (D2B12 XP Rabbit mAb Chip formulated, Cell Signaling Technology) and the normal goat IgG control (R&D Systems, cat. AF3639) as a negative control. After immunoprecipitation, the DNA was isolated using spin columns from the kit and eluted in 50 μL Elution Reagent C. PCR was performed with 2 µL of immunoprecipitated material and the products were analyzed on an 1.5% agarose gel, and visualized using a gel documentation system.

The following primers were used to quantify SNAIL binding to the *EZRIN* promoter fragments described in previous section:

*EZR_1* fragment forward primer: 5′-GAGGCTAGCACGAGTTAAGCA-3′
*EZR_1* fragment reverse primer: 5′-GCACGTTTGTGGCCTCTTTT-3′
*EZR_2* fragment forward primer: 5′-GGAGCACACGGAGCACTG-3′
*EZR_2* fragment reverse primer: 5′-CGGAGAGAGGCGGAGAAGA-3′

Additionally, Chip-Seq analysis was performed using the Intelliseq sp. z o. o. (Cracow, Poland) company service in cooperation with Novogene Co., Ltd (Cambridge, UK) using Illumina high-throughput sequencer. Quality assessment of the reads was performed using the FastQC tool (v0.11.7). Reads containing adapters were filtered using the Trim Galore tool (v0.5.0). Mapping of reads to the human reference genome GRCh38 from the Ensembl database was done using the BWA-MEM software (v. 2.1.0). The aligned reads were filtered out more than once. Subsequently, the analysis using the MASC2 callpeak tool (v. 2.1.2) with default parameters (minimum FDR 0.05) was performed. The detected transcription factor binding sites were noted using the HOMER annotatePeaks.pl tool (v4.10) and a GTF file from the Ensembl database. The Integrative Genomics Viewer (IGV) was used as a visualization tool for interactive exploration of large, integrated genomic datasets [61]. ChIP-seq data data were deposited in Gene Expression Omnibus (GEO) under accession number GSE152355.

### 4.14. Scratch Assay

Confluent RMS cells were treated with DMEM medium with 0.5% BSA for 24 h. Subsequently, a scratch was generated with a pipette tip. Starving medium was replaced every day. Photographs were taken after 24, 48 and 72 h and they were analyzed using ImageJ software (National Institutes of Health, Bethesda, MD, USA).

### 4.15. Chemotaxis and Invasion Assays

Chemotaxis of RMS cells to 20 ng/mL HGF (R&D System) and 100 ng/mL SDF-1 (Peprotech, Rocky Hill, NJ, USA) was evaluated using modified Boyden’s chamber with 8 μm pore polycarbonate membrane inserts (Transwell; Corning Life Sciences—PZ HTL SA, Warsaw, Poland), as described previously [21]. 0.5% BSA served as a negative control. Similarly, invasion of RMS cells through growth factor reduced Matrigel invasion inserts (Corning Life Sciences) to 10% FBS, 20 ng/mL HGF, 100 ng/mL SDF-1, 0.5% BSA was also investigated, as described previously [21].

### 4.16. Immunofluorescent Staining with Phalloidin

RH30 cells were fixed in 4% formaldehyde (POCH, Gliwice, Poland) in PBS, permeabilized in 0.1% TritonX-100 (Sigma-Aldrich), blocked in 1% bovine serum albumin (BSA, Sigma-Aldrich). For visualization of the actin cytoskeleton, the cells were stained with phalloidin conjugated with Alexa Fluor 488 (Life Technologies) according to the manufacturer’s protocol. Labeling was assessed by fluorescence microscopy using an Olympus BX51 or IX70 microscope (Olympus Corporation, Tokyo, Japan) and Olympus XC50 camera with cellSens Dimension software (both from Olympus). The images were processed using cellSens Dimension software.

### 4.17. Flow Cytometry

For evaluation of MET and CXCR4 receptors expression levels RMS cells were stained with monoclonal FITC-labeled anti-human HGFR/c-MET antibody, clone 95106 (R&D), PE-labeled anti-human CXCR4 antibody (Becton Dickinson, Franklin Lakes, NJ, USA) or mouse IgG1 isotype control (R&D) labeled with FITC or PE respectively. The cells were acquired using FACS Canto II cytometer (Becton Dickinson) and analyzed using FACS Diva software (Becton Dickinson), as described previously [21].

The expression level of adhesion molecules was evaluated using Lyoplate technology (Lyoplate Screening Panel, Becton Dickinson) according to the manufacturer’s protocol. The cells were acquired by use of Attune Next Flow Cytometer and analyzed using Attune NxT Software v2.2 (Thermo Fisher Scientific, Waltham, MA, USA).

### 4.18. Adhesion Assay

HUVEC endothelial cells (5 × 10^4^ per well) were seeded in black 96-well plates with a clear bottom (Corning Costar, Amsterdam, The Netherlands) and grown overnight to a confluent monolayer. After stimulation of endothelial cells with TNF-α (50 ng/mL) for 24 h, RMS cells were incubated with 2.5 μM Calcein AM (BD Pharminogen, Franklin Lakes, NJ, USA) for 30 min at 37 °C in cell culture medium, washed, then they rested for 30 min and treated with 100 ng/mL SDF-1 for 15 min or 20 ng/mL HGF for 30 min or they were in control medium without factors. 1 × 10^4^ RMS cells were added to the endothelial monolayers and incubated at 37 °C for 15 min. Plates were washed three times with phosphate–buffered saline to remove unbound cells and the fluorescence was read using a fluorescence plate reader (Spark™ 10M multimode microplate reader, Tecan, Männedorf, Switzerland) with excitation at 495 nm and emission intensity detected at 515 nm. The results were normalized to percentage of the cells in control conditions.

### 4.19. In Vivo Experiments

Animal experiments were approved by the Local Ethics Committee in Krakow (no 23/2013). To study metastasis, 1 × 10^6^ RH30 cells were implanted intravenously into immunodeficient NOD-SCID mice for 7 days. Each experimental group contained five animals, and all of the experiments were repeated three times. The appearance of RMS cells in the lungs and in the bone marrow samples from the left and right legs was evaluated by real-time PCR using human GAPDH specific primers-probe set (Hs99999905_m1; Applied Biosystems) compared to murine GAPDH (Mm99999915_g1; Applied Biosystems) using ΔC_t_ method. The level of SDF-1 in murine lungs was evaluated by real-time PCR using murine SDF-1 primers-probe set (Mm00445552_m1; Applied Biosystems) compared to murine GAPDH (Mm99999915_g1; Applied Biosystems) using ΔC_t_ method.

### 4.20. Bioinformatical Analysis of Microarray Data from RMS Patients

For gene expression analysis in a group of 158 RMS patients we used data from GEO database, stored under accession number GSE92689 [62]. Background subtraction and data normalization was performed with affy [63] package in R/Bioconductor and the average expression was used for further statistical analysis. Pearson correlation of gene expression was analyzed using GraphPad Prism software (GraphPad Software, San Diego, CA, USA).

### 4.21. Statistical Analysis

Unless stated otherwise, the results show the mean ± standard error of the mean (SEM) of at least 2 to 4 independent biological experiments, as stated in figure legends (*n* value). Statistical analysis was performed by one-way analysis of variance (ANOVA) with Tukey or Dunnett’s post-test or Student’s *t*-test using GraphPad Prism software. Differences with a *p*-value less than 0.05 were considered statistically significant.

## 5. Conclusions

Here, we demonstrate for the first time that SNAIL stimulates the metastatic abilities of RMS cells both in vitro and in vivo. We propose a novel mechanism of its action that may be important in other tumor types. We postulate that, EZRIN, as a protein mediating between plasma membrane and cytoskeleton, PI3K-AKT signaling and miRNAs link SNAIL with the RMS cell migratory machinery.

## Figures and Tables

**Table 1:** miRNA sequencing results revealed miRNAs that were significantly differentially expressed between RH30 shSNAIL and shCTRL cells. The table shows the log fold change (logFC) between the shSNAIL and shCTRL groups; raw *p*-values, Benjamini-Hochberg FDR corrected *p*-values; *n* = 3.

Names	logFC	*p* Value	FDR
hsa-miR-1269b	−11.78	1.12 × 10^−83^	6.95 × 10^−82^
hsa-miR-218-5p	−11.07	1.50 × 10^−142^	2.10 × 10^−140^
hsa-miR-548h-5p	−10.81	5.84 × 10^−61^	1.82 × 10^−59^
hsa-miR-452-5p	−10.73	3.40 × 10^−174^	1.47 × 10^−171^
hsa-miR-224-5p	−10.68	1.22 × 10^−40^	1.89 × 10^−39^
hsa-miR-3148	10.32	9.71 × 10^−56^	2.82 × 10^−54^
hsa-miR-4652-5p	−9.78	7.14 × 10^−43^	1.24 × 10^−41^
hsa-miR-1269a	−9.77	6.90 × 10^−156^	1.51 × 10^−153^
hsa-miR-873-5p	−9.63	5.99 × 10^−41^	9.65 × 10^−40^
hsa-miR-873-3p	−8.79	1.56 × 10^−53^	4.25 × 10^−52^
hsa-miR-302a-5p	−8.07	8.53 × 10^−45^	1.69 × 10^−43^
hsa-miR-105-5p	−6.72	2.21 × 10^−61^	7.38 × 10^−60^
hsa-miR-139-5p	6.53	2.01 × 10^−65^	8.75 × 10^−64^
hsa-miR-199b-5p	−6.52	4.51 × 10^−94^	3.27 × 10^−92^
hsa-miR-95-3p	6.42	2.34 × 10^−40^	3.51 × 10^−39^
hsa-miR-767-5p	−6.15	8.59 × 10^−51^	1.97 × 10^−49^
hsa-miR-139-3p	5.93	1.25 × 10^−44^	2.37 × 10^−43^
hsa-miR-28-5p	5.43	1.15 × 10^−94^	1.00 × 10^−92^
hsa-miR-143-3p	−5.26	2.17 × 10^−53^	5.55 × 10^−52^
hsa-miR-28-3p	5.1	3.60 × 10^−113^	3.88 × 10^−111^
hsa-miR-193a-5p	4.45	6.18 × 10^−75^	3.36 × 10^−73^
hsa-miR-541-3p	−4.32	4.72 × 10^−63^	1.71 × 10^−61^
hsa-miR-412-5p	−4.15	4.11 × 10^−71^	1.99 × 10^−69^
hsa-miR-1197	−3.83	1.45 × 10^−50^	3.15 × 10^−49^
hsa-miR-200c-3p	−3.77	1.50 × 10^−64^	5.95 × 10^−63^
hsa-miR-431-5p	−3.76	5.00 × 10^−45^	1.04 × 10^−43^
hsa-miR-200b-3p	−3.65	3.26 × 10^−41^	5.45 × 10^−40^
hsa-miR-129-5p	−3.32	2.27 × 10^−43^	4.11 × 10^−42^
hsa-miR-1180-3p	−3.27	4.16 × 10^−51^	1.01 × 10^−49^
hsa-miR-486-5p	2.74	9.05 × 10^−40^	1.31 × 10^−38^

## Supplementary Materials

The following are available online at https://www.mdpi.com/2072-6694/12/7/1870/s1. Table S1. Table presenting SNAIL binding sites in RH30 cells evaluated by ChIP-seq. Table S2. Table presenting the results from next generation sequencing of miRNAs in RH30 WT, shCTRL and shSNAIL cells. Figure S1. SNAIL expression in RH30 cells after transduction with lentiviral vectors encoding shRNA. Figure S2. The effect of SNAIL silencing on motility of RH30 cells in a scratch assay. Figure S3. SDF-1 level in murine lungs. Figure S4. SNAIL and EZRIN levels after transfection of RMS cells with siRNA. Figure S5. Genome browser views of SNAIL binding to AKT2 promoter and its own promoter (ChIP-seq data). Figure S6. Supplementary Western blot images—images of the whole uncropped membranes with ladder.
