# Supplementary material for: SNAIL Promotes Metastatic Behavior of Rhabdomyosarcoma by Increasing EZRIN and AKT Expression and Regulating MicroRNA Networks
Source: Cancers (Basel). 2020 Jul 11;12(7):1870. doi: 10.3390/cancers12071870 (PMC7408994; doi:10.3390/cancers12071870)
Supplement: Supplementary file 1 [file cancers-12-01870-s001.zip › Supplementary Figures.pdf]

SNAIL Promotes Metastatic Behavior of Rhabdomyosarcoma by Increasing EZRIN and AKT Expression and Regulating MicroRNA Networks

Klaudia Skrzypek, Marta Kot, Paweł Konieczny, Artur Nieszporek, Anna Kusienicka, Małgorzata Lasota, Wojciech Bobela, Urszula Jankowska, Sylwia Kędracka-Krok and Marcin Majka

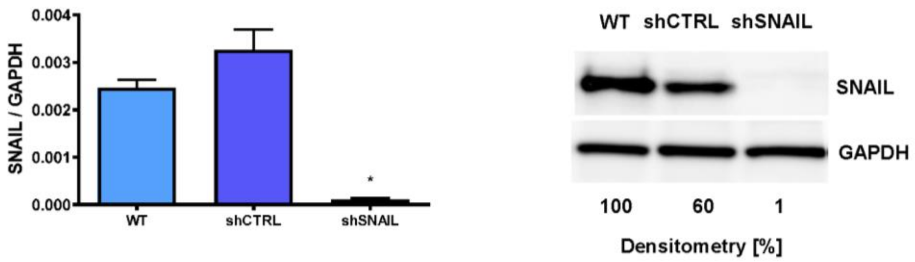

Supplementary Figure S1. SNAIL expression in RH30 cells after transduction with lentiviral vectors encoding shRNA. SNAIL was downregulated at the mRNA and protein levels after transduction of RH30 cells with shRNA lentiviral vectors targeting SNAIL (shSNAIL) and selection with puromycin. SNAIL silencing was validated by qPCR (n = 3; *p<0.05.) and Western blotting (representative image). Graphical data are presented as means +/− SEMs.

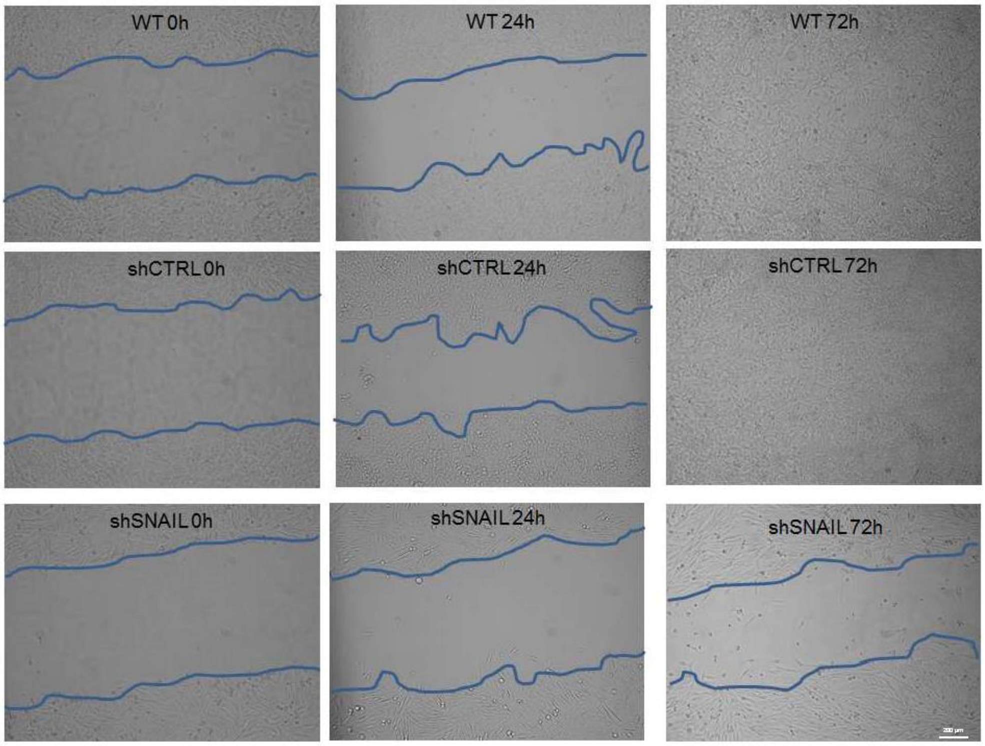

Supplementary Figure S2. The effect of SNAIL silencing on motility of RH30 cells in a scratch assay. The images shows representative photos of scratch assay experiments. SNAIL deficient RH30 cells (shSNAIL) closed the gap in a scratch assay slower than control (shCTRL) and wild-type (WT) cells. Blue lines show the edges of scratches. White scale bar represents 200 μm.

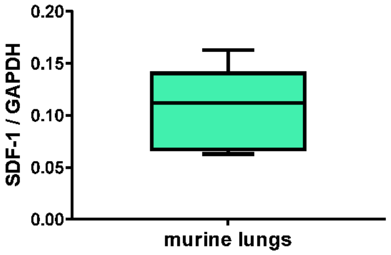

Supplementary Figure S3. SDF-1 level in murine lungs. SDF1 was expressed in murine lungs; whisker min to max plot; qPCR calculated with DCt method using GAPDH as a relative control (Ct value for SDF-1 was 29.64 +/- 0.60, whereas for GAPDH it was 26.30 +/- 0.42), $n = 5$.

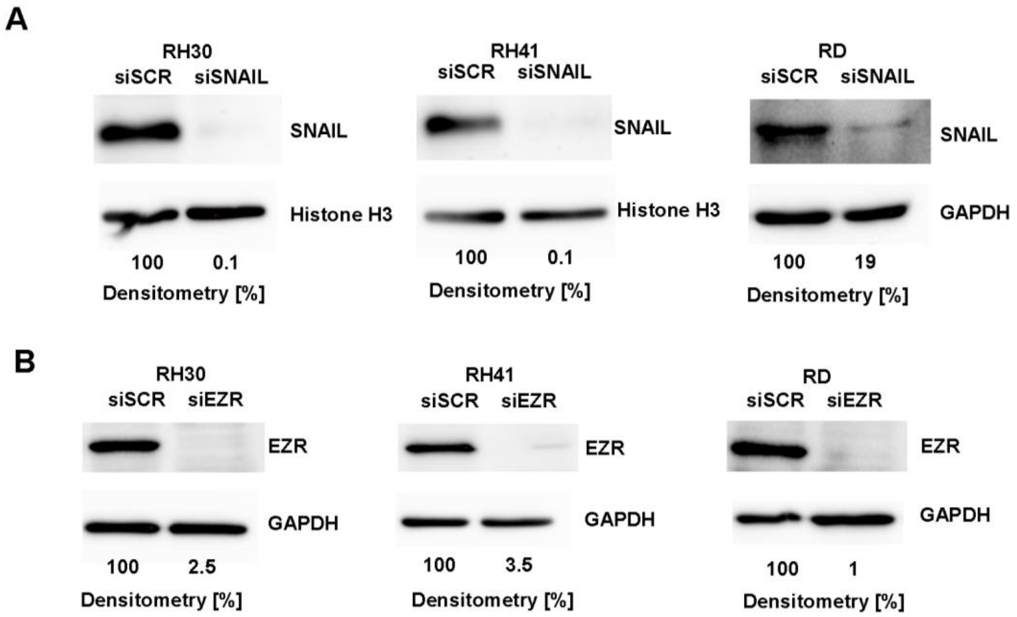

Supplementary Figure S4. SNAIL and EZRIN levels after transfection of RMS cells with siRNA. (A) SNAIL protein expression was strongly downregulated after transfection of RH30, RH41 and RD cells with siRNA (representative Western blot images). **(B)** EZRIN protein expression was strongly downregulated after transfection of RH30, RH41 and RD cells with siRNA (representative Western blot images). Densitometric analysis evaluated the ratio of EZRIN/GAPDH and was presented as percentage of control.

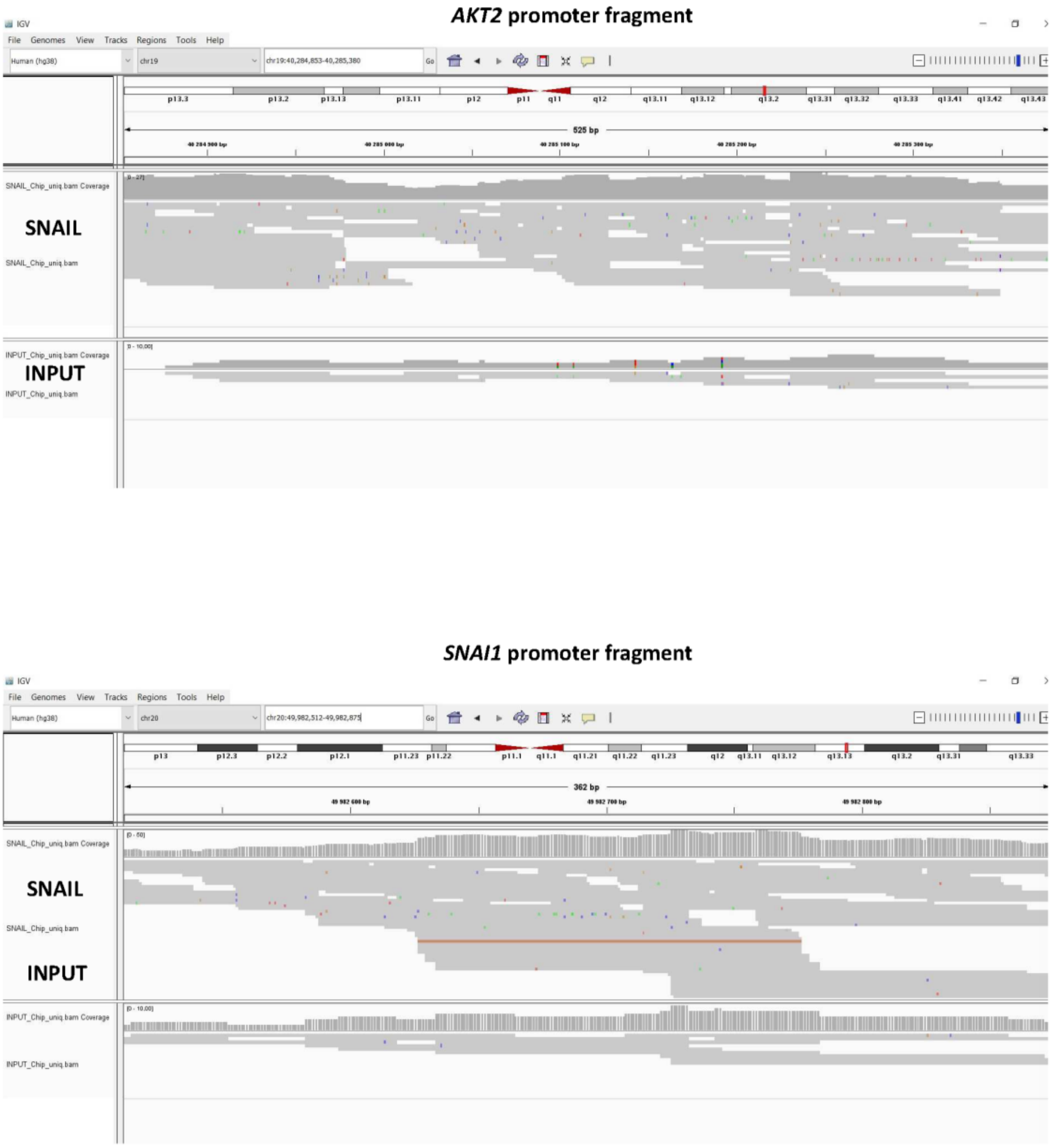

Supplementary Figure S5. Genome browser views of SNAIL binding to AKT2 promoter fragment and its own promoter fragment (ChIP-seq data). The Integrative Genomics Viewer (IGV) was used as a visualization tool for interactive exploration of large, integrated genomic datasets from ChIP-seq results.

Fig. 2C – Western blot images

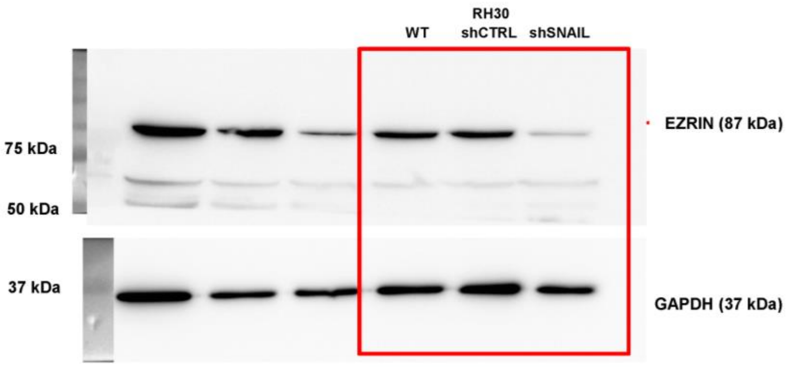

2D – RH41 – Western blot images

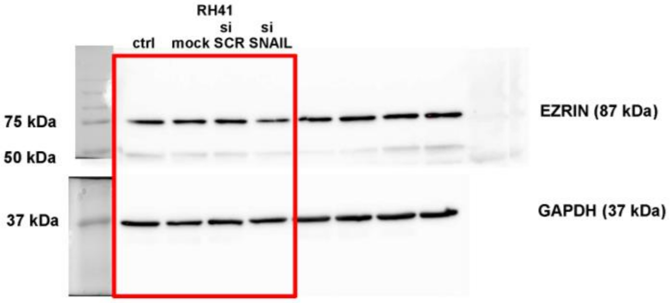

2D – RD – Western blot images

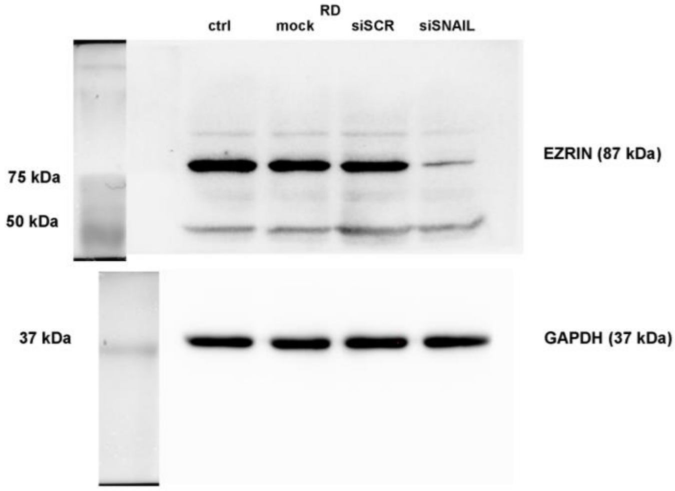

Figure S6. *Cont.*

Fig. 3H – Western blot images

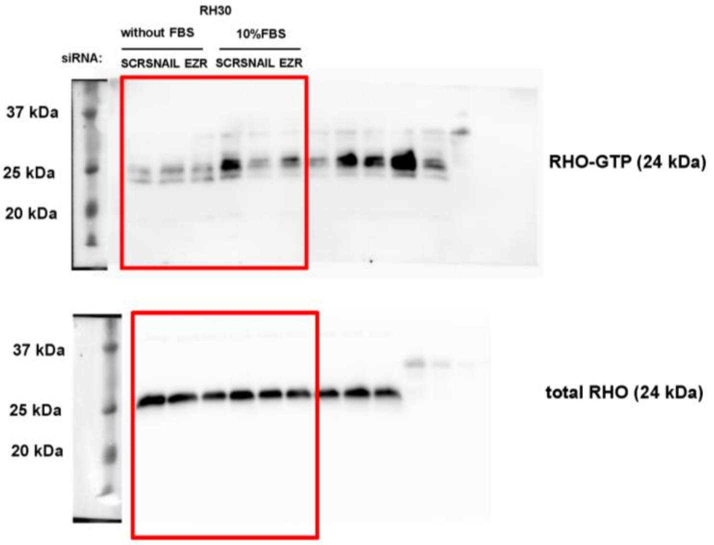

Fig. 4B– Western blot images

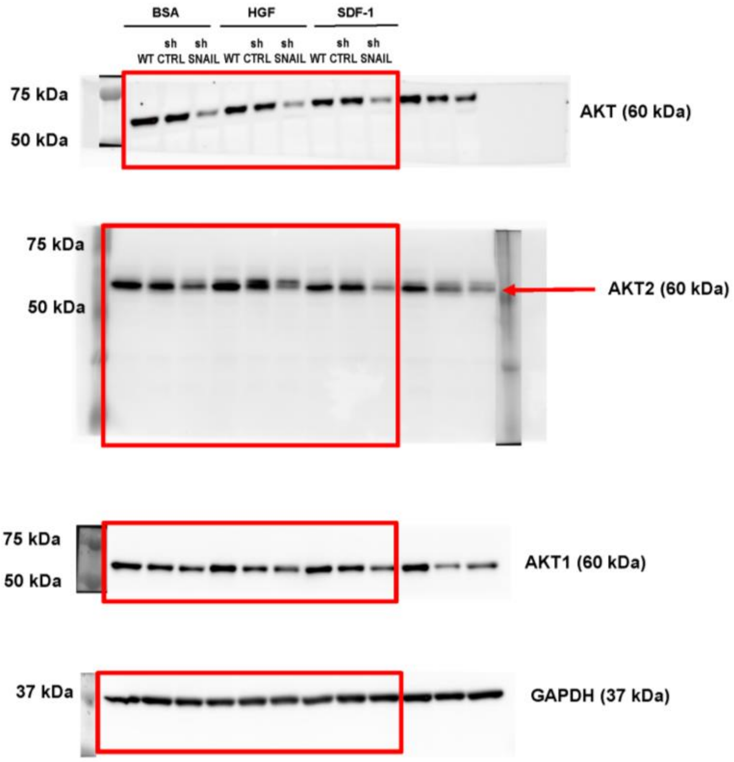

Figure S6. *Cont.*

Fig. 4C – Western blot images

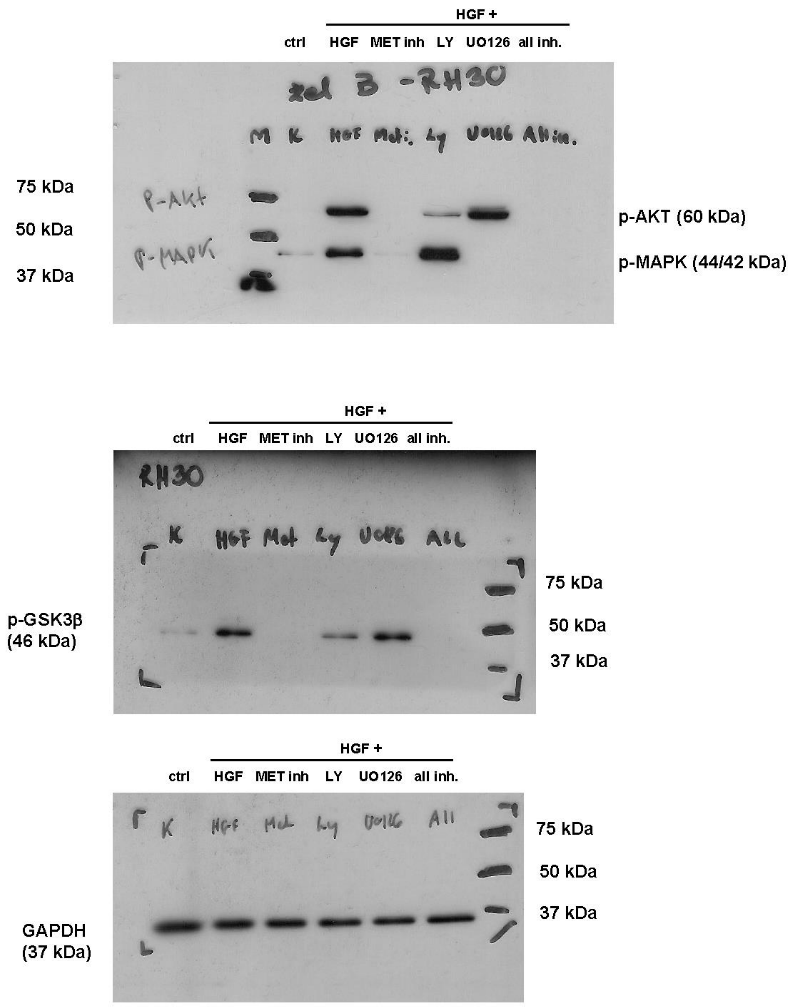

Figure S6. *Cont.*

Fig. 4D – Western blot images

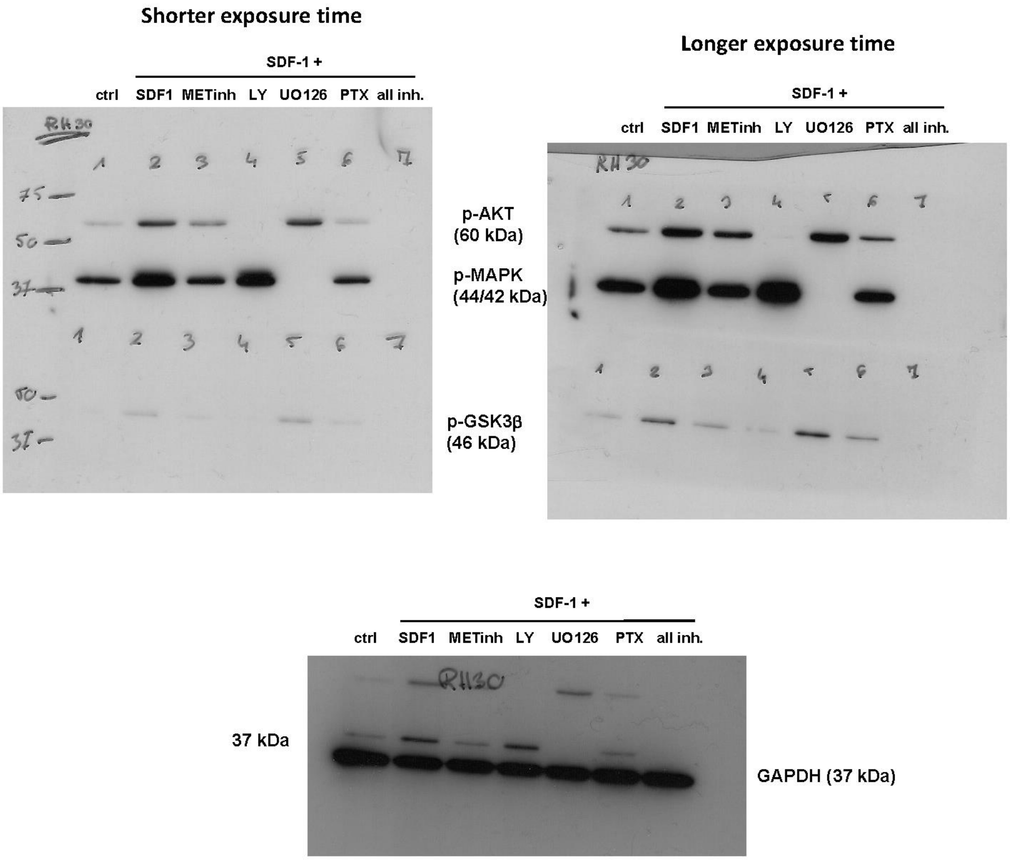

Fig. 4E – Western blot images

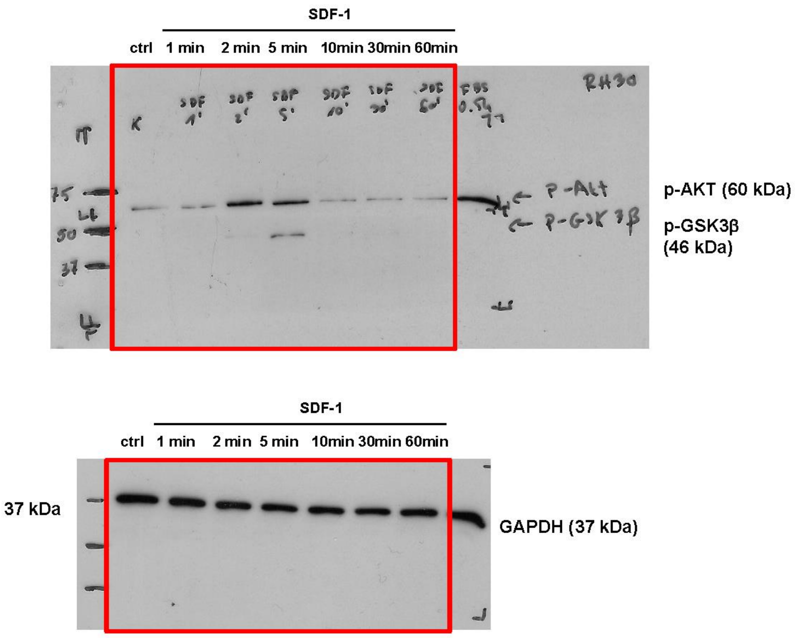

Figure S6. *Cont.*

Fig. 4F – Western blot images

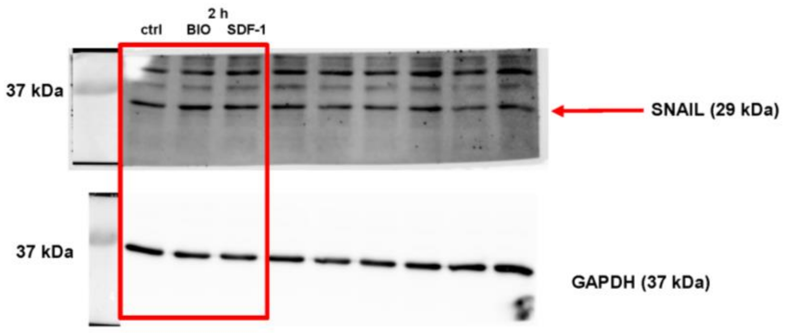

Fig. 4G – Western blot images

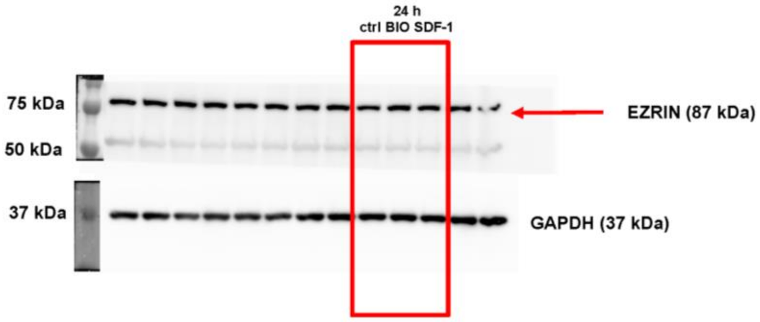

Fig. 5A – Western blot images

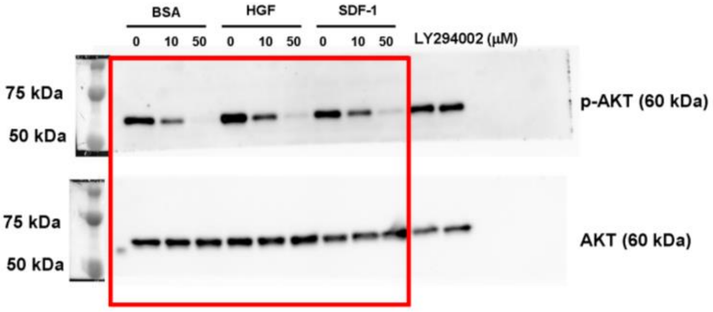

Figure S6. *Cont.*

Fig. 7D – Western blot images

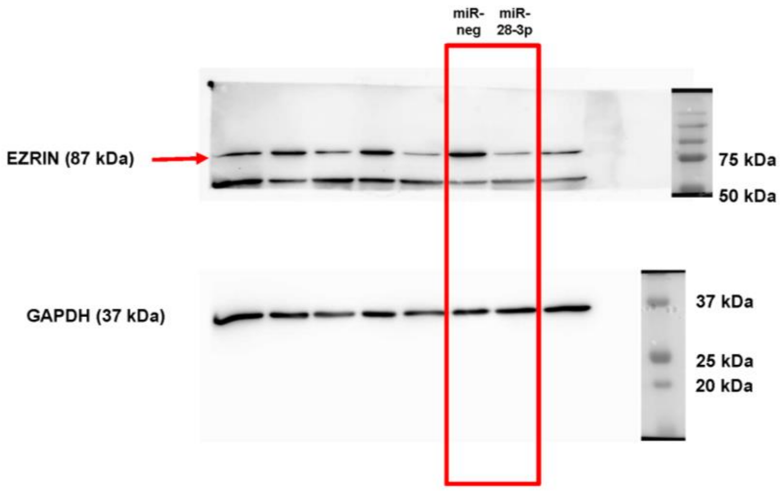

Supplementary Fig. S1 – Western blot images

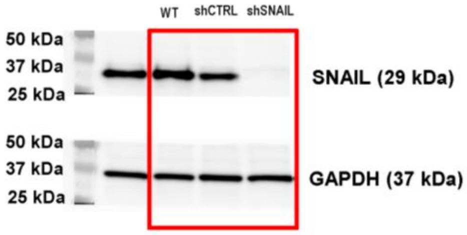

Figure S6. *Cont.*

Supplementary Fig. S4A– Western blot images

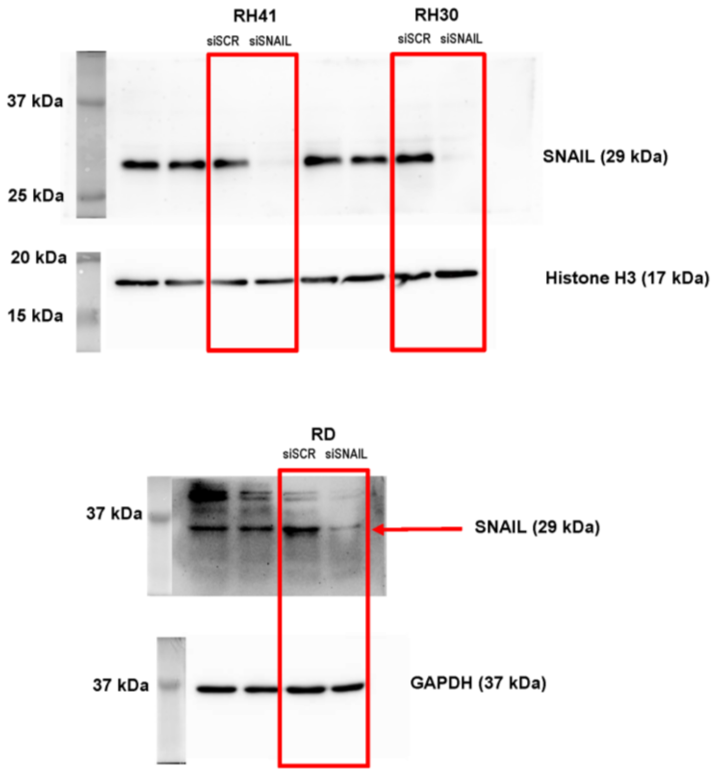

Supplementary Fig. S4B – Western blot images

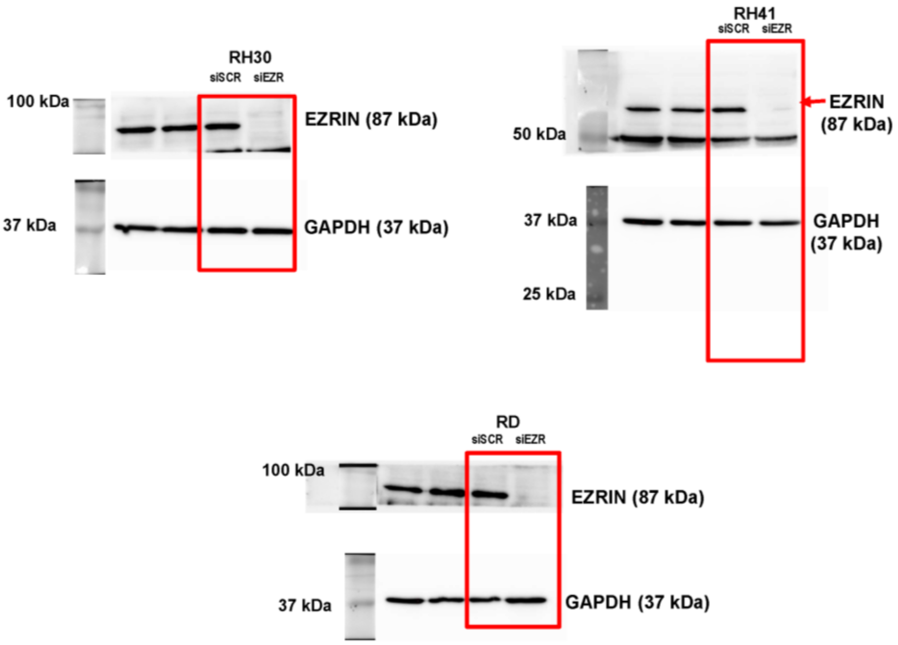

Supplementary Figure S6. Supplementary Western blot images – images of the whole uncropped membranes with ladder. The images are presented on the separate pages.

© 2020 by the authors. Licensee MDPI, Basel, Switzerland. This article is an open access article distributed under the terms and conditions of the Creative Commons Attribution (CC BY) license (http://creativecommons.org/licenses/by/4.0/).
